# Absorption Correction for Reliable Pair Distribution Functions from Low Energy X‑ray Sources

**DOI:** 10.1021/acs.cgd.5c00551

**Published:** 2026-01-21

**Authors:** Yucong Chen, Till Schertenleib, Andrew Yang, Pascal Schouwink, Wendy L. Queen, Simon J. L. Billinge

**Affiliations:** † Department of Applied Physics and Applied Mathematics, 5798 Columbia University, New York, New York 10027, United States; ‡ Institute of Chemical Sciences and Engineering (ISIC), 27218 École Polytechnique Fédérale de Lausanne (EPFL), Sion CH-1951, Switzerland

## Abstract

This paper explores the development and testing of a simple absorption correction model for processing powder X-ray diffraction data from Debye–Scherrer geometry laboratory X-ray experiments. This may be used as a preprocessing step before using PDFgetX3 to obtain reliable pair distribution functions (PDFs). Various experimental and theoretical methods for estimating μ*R* were explored, and the most appropriate μ*R* values for correction were identified for different capillary diameters and X-ray beam sizes. We identify operational ranges of μ*R* where a reasonable signal-to-noise ratio is possible after correction. A user-friendly software package, diffpy.labpdfproc, is presented that can help estimate μ*R* and perform absorption corrections with a rapid calculation for efficient processing.

## Graphical abstract

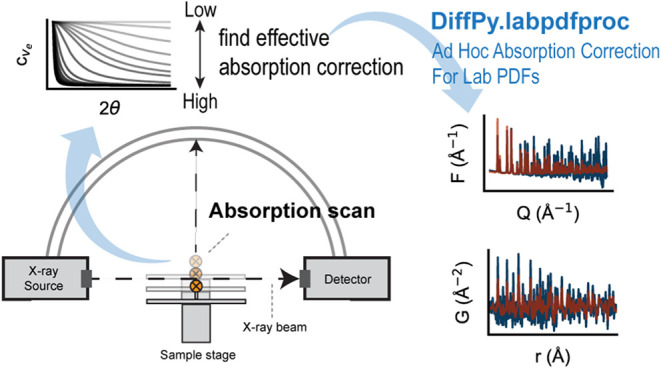

## 1 Introduction

Historically, the study of local atomic structures using X-ray pair distribution function (PDF) analysis has predominantly been done on data from synchrotron facilities using rapid acquisition PDF (RAPDF) techniques.^1^ However, using laboratory-based X-ray sources to get good PDFs is of great interest. This has been demonstrated from laboratory diffractometers equipped with Mo and Ag *K*_α_ sources in various examples, including crystalline materials^2^ and nanoparticles.^3^

The current authors recently tested protocols for optimizing experimental setups for such measurements.^4^ That work discussed that some factors affecting the data reduction to obtain quantitatively accurate PDFs that are often neglected in RAPDF experiments become more relevant for lab-based experiments, especially for those done with Mo *K*_α_ radiation. In particular, multiplicative corrections to the measured intensities from sample absorption are likely to have a non-negligible *Q*-dependence for Mo *K*_α_ data but not for RAPDF data.^4,5^ This occurs for two reasons. First, the lower X-ray energies result in overall higher sample absorption effects. Second, in lab experiments, it is necessary to measure over a large range of 2θ, typically up to 140° or higher with Mo or Ag X-rays, making the intensities more susceptible to any angle-dependent multiplicative corrections.

The influence of sample absorption has been discussed for powder diffraction in general. Absorption leads to differences in calculated and measured powder diffraction patterns and has to be accounted for in Rietveld refinements.^6,7^ In Bragg–Brentano geometries finite sample thickness effects must be considered when measuring low absorbing specimens.^5,8^ Absorption effects are also crucial in successful quantitative XRD analysis by multiphase refinements.^9–11^ Here, we present a detailed investigation of the influence of sample absorption in benchtop PDF analysis, i.e., for lab diffractometers with capillary (Debye–Scherrer) geometry. We present a software package for making sample absorption corrections to measured data for this geometry.

Among the numerous available software packages to obtain *G*(*r*) from raw data,^12–17^ PDFgetX3 ^18^ is widely used by the community as it follows a simple *ad hoc* approach to data reduction.^19^ The *ad hoc* algorithm used in PDFgetX3 does a good job of correcting for parasitic scattering (unwanted additive contributions to the signal) but makes no correction for multiplicative effects. This works well for RAPDF data, where they are small, and their angle dependence is even smaller. However, as discussed above, when applied to data from a laboratory diffractometer, this simplifying assumption may not be valid in general, which motivated this study.

This work also allows us to suggest best practices for sample preparation for PDF experiments on laboratory X-ray diffractometers to mitigate the worst effects of sample absorption. These strategies are not new, but here, we validate and quantify them somewhat to give more precise guidance.

## 2 Historical Context

The *International Tables of Crystallography* ^20^ serves as a benchmark for absorption corrections. Earlier studies focused on developing more accurate models for these corrections, while later works emphasized faster computations, additional correction factors, or software implementations. Therefore, we also compare our calculations with the results presented there in Supporting Information Section 3. In large part, software implementations of the corrections are applied to models fit to the data and not applied to the data itself, for example, in Rietveld fits. In the context of PDF analysis, the approach of applying the corrections directly to the data is conventionally followed, and our software diffpy.labpdfproc does this.

Foundational work on absorption corrections includes Claassen,^21^ which presents a short derivation showing why the absorption factor *A* can be expressed in terms of μ*R*, along with an estimation of correction factors for heavy powders with an application to experimental data. Bradly^22^ devised a method for calculating the absorption factor that may be used for all, but especially larger, values of μ*R*, and raised the point that combining it with Claassen’s graphical method minimizes the error. Bond^23^ introduced an absorption correction for cylindrical samples on equi-inclination Weissenberg cameras that worked for μ*R* ≤ 8. Albrecht^24^ presented a graphical method for corrections in crystals regardless of their size, shape, or absorbing power. Early work on weakly scattering amorphous or liquid samples contained in cylindrical containers, where the absorption of the contained is non-negligible compared to that of the sample, was done by Ritter et al., using graphical methods described by Claasen. It was shown that in the case of sufficiently thin containers, the angle dependence of the container contribution is negligible, and rather, can be approximated by an overall uniform reduction of signal intensity.^25^ Later, Paalman and Pings^26^ presented a numerical approach to solve the integrals, providing a computationally faster solution to the graphical methods of Ritter et al.^25^ This approach was later generalized by Kendig and Pings^27^ to also handle situations where the incident X-ray beam is smaller than the sample container. Dwiggins^28^ provided exact analytical solutions (direct integration) for 0 and 90° and evaluated accuracy up to μ*R* = 5, and later proposed a rapid calculation for cylinders to an accuracy of 0.1%.^29^ Paalman and Pings, and Dwiggins evaluate the same quantity and integrals, but use different numerical approaches formulated in different base coordinates. Dwiggins’ fast approach covers only small angular ranges (0° ≤ 2θ ≤ 90°) and makes use of simple equations that make interpolations between precomputed tabulated values. On the other hand, Paalman and Pings provide a general solution covering a larger angular range and allowing the combined or separate evaluation of sample and container contribution. Current popular programs used to extract PDFs from XRD data, such as GudrunX and PDFgetX2, use the approach outlined by Paalman and Pings and Kendig and Pings to compute absorption corrections, largely because of the need to cover a larger angular range to maximize the covered *Q*-range.

More recent studies have continued to advance the field. For example, Sabine et al.^6^ derived analytical expressions for Bragg peak shifts due to absorption effects, and tested them across both small and large μ*R*’s. Ida^30^ compared the efficiency of Thorkildsen and Larsen’s method with that of Dwiggins, finding the former to be more efficient. Finally, Coelho and Rowles^7^ focused mostly on addressing peak shifts, describing capillary specimen aberrations for X-ray powder diffraction line profiles for various beam geometries. Meanwhile, software implementations such as GSAS-II,^15^ FullProf,^31^ and TOPAS^17^ have incorporated these corrections into Rietveld refinement models. For example, GSAS-II contains an approximation of correction results with errors within 0.2–0.5%. GSAS-II and TOPAS can also produce *G*(*r*) from powder diffraction data, and their implemented absorption corrections can be applied to the raw data. However, the effect of this on PDF refinements has not been explored in detail.

Current literature has been thorough in developing accurate correction formulas and various applications. Our goal is not to outperform these existing approaches or to propose a new method. In contrast, our focus is to provide an educational derivation aimed at building an intuitive understanding of the absorption correction process. To our knowledge, such a complete derivation is not present explicitly in the literature, except for a brief version in *The International Tables of Crystallography* ^32^ and Albrecht.^24^ Here, we expand and present a complete derivation based on brute force computation in Supporting Information Section 2. In addition, we introduce a fast interpolation method for computing the corrections given any arbitrary μ*R* values, allowing users to quickly test different μ*R* values without recomputing the correction curve every time. Finally, in the context of corrections to data for PDF analysis, we explore how applying these corrections affects the data, which has not been systematically studied before, as previously the emphasis was on applying corrections to models in the context of Rietveld refinement rather than applying corrections directly to the data. To help users build intuition, we analyze the effect of the corrections on model fits to the resulting PDFs.

## 3 Absorption Correction

Here, we address the multiplicative correction due to sample self-absorption effects for the most common geometry used for PDF analysis, the Debye–Scherrer geometry, in which a beam is incident on a cylindrical sample in a capillary with a detector rotating around the sample. As described above, the absorption correction for this geometry has been developed in detail over many years and is routinely applied in neutron PDF measurements. Various codes implement these corrections as part of the total scattering data reduction workflow.^7,14,33,34^ They require rather detailed knowledge of the sample composition and packing fraction and, for the greatest accuracy, also the sample and sample container geometry.

In this work, we are more concerned with obtaining sufficient accuracy in these corrections in combination with *ad hoc* data reduction approaches used as PDFgetX3, and understanding the effects of approximate corrections on resulting PDFs. We explore this for a range of possible sample compositions with Mo *K*_α_ and Ag *K*_α_ radiation. We first briefly describe a simplified derivation of the angle dependence of the absorption in this geometry and then explore the nature of the resulting curves in different situations. The full derivation is in the Supporting Information, but is summarized here, as it can help to build intuition about absorption effects in a laboratory X-ray setting.

Assuming a homogeneous ideal powder in the absence of sample absorption, the measured X-ray intensity at some point in *Q*, *I*_m_(*Q*), would be proportional to the illuminated sample volume, *V*. Here, *Q* is the magnitude of the scattering vector, $Q=\left|k_{i}-k_{s}\right|=\frac{4\pi \sin\theta }{\lambda }$ , where **k**_*i*_ and **k**_s_ are the incident and scattered wave vectors, respectively, θ is the Bragg angle which is half the scattering angle, 2θ, which is the angle between the incident and scattered X-ray beams (see Figure 1), and λ is the X-ray wavelength. A normalized coherent scattering intensity per unit volume of the sample, *i*_c_, can therefore be obtained by dividing *I*_m_ by *V*.

**Figure 1:**
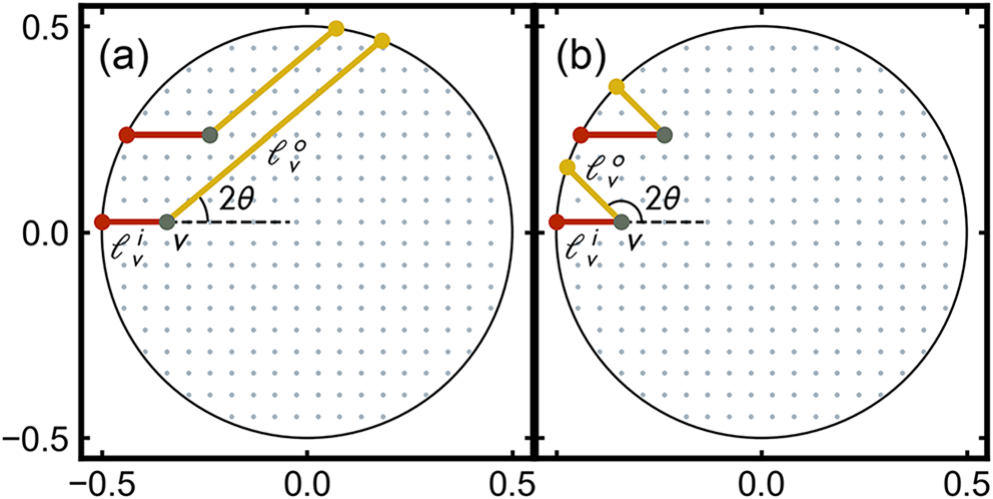
Incoming and outgoing X-ray beam paths for X-rays undergoing scattering in two representative pixels at an angle (a) 2θ = 40° and (b) 2θ = 135°. In each case, the large black circle indicates the edge of a cross section of the cylindrical sample capillary. The X-rays arrive horizontally from the left. A scattered ray is then shown at the given 2θ angle. The small blue-gray dots indicate positions of a uniform grid of voxels in the circle, and the red and yellow dots indicate the point on the surface of the sample where the X-rays enter and exit, respectively. The path length for the X-ray scattered in the *v*th voxel at angle 2θ is then $l_{v}\left(2\theta \right)=l_{v}^{i}+l_{v}^{o}\left(2\theta \right)$ , the sum of the lengths of the red line and the yellow lines.

In the presence of sample absorption, the scattered intensity from any small volume element (voxel) in the sample will be reduced by absorption of the X-ray as it travels along the incoming, $l_{v}^{i}$ , and outgoing, $l_{v}^{o}$ , path through the sample. This is shown in Figure 1 for the case of a cylindrical capillary mounted perpendicular to the beam. The derivation of the path lengths is reproduced in detail in Section S1 in the Supporting Information. When there is significant sample absorption, to get *i*_c_, we would divide *I*_m_ not by the full illuminated volume but by an absorption-corrected effective volume, *V*_e_, so that

1 $$i_{c}\left(2\theta \right)=\frac{I_{m}\left(2\theta \right)}{V_{e}\left(2\theta \right)}$$

where the effective volume is given by^4^

2 $$V_{e}\left(2\theta \right)=\sum_{v}e^{-\mu_{s}^{\lambda }l_{v}\left(2\theta \right)}\Delta V_{v}$$

where Δ*V*_*v*_ is the volume of the *v*th voxel and μ_*s*_^λ^ is the linear absorption coefficient of the sample for X-rays of wavelength λ. $l_{v}\left(2\theta \right)$ is the total path length, i.e., the sum of the incident and outgoing path lengths (see Figure 1)­

3 $$l_{v}\left(2\theta \right)=l_{v}^{i}+l_{v}^{o}\left(2\theta \right)$$

for the *v*th voxel and scattering angle 2θ. The normalized intensity from eq 1 is then

4 $$i_{c}=\frac{I_{m}}{\sum_{v}e^{-\mu_{s}^{\lambda }l_{v}}\Delta V_{v}}.$$

For the case where all of the voxels have the same volume Δ*V*_*v*_ = *V*/*N*_*v*_, we get

5 $$i_{c}=\frac{N_{v}I_{m}}{V\sum_{v}e^{-\mu_{s}^{\lambda }l_{v}\left(2\theta \right)}}$$

We can then define an absorption correction A* as

6 $$A^{*}\left(2\theta \right)=\frac{N_{v}}{\sum_{v}e^{-\mu_{s}^{\lambda }l_{v}\left(2\theta \right)}}$$

Both the strength of the X-ray attenuation and its angle dependence are dependent on the material and wavelength-specific linear absorption coefficient, μ_*s*_^λ^. They also depend on a sum over all of the path lengths an X-ray takes through the sample as it arrives from the source and exits after scattering.

For simpler flat geometries, it is known that the curve shape depends only on the product μ*t*, where *t* is the thickness of the sample, and not independently on μ and *t*. We show here that this is also true for the capillary geometry: the curve depends only on the product μ*R*, where *R* is half of the capillary diameter. This greatly simplifies the analysis by reducing the dimensionality of the space of possibilities that we need to consider. Details of the proof are provided in Section S2 in the Supporting Information. There, we show that μ*R* can be factored out of the sum, $\sum_{v}e^{-\mu_{s}^{\lambda }l_{v}\left(2\theta \right)}$ in eq 7, which can be rewritten as

7 $$A^{*}\left(2\theta \right)=\frac{N_{v}}{\sum_{v}e^{-\mu_{s}^{\lambda }R\cdot dl_{v}\left(2\theta \right)}}$$

where $dl_{v}\left(2\theta \right)$ is the path length that the X-ray would traverse for a capillary of unit diameter. Thus, the absorption correction depends not on μ and *R* independently but on the product μ*R* (where, for compactness, we drop hereafter the superscript and subscript that explicitly indicate that it is μ*R* for the sample at a particular wavelength). To obtain the absorption-corrected data, we use *I*_m_ × A*.

We explore below the effect on the PDF of applying this correction to data from a variety of samples of different absorptions, discuss different ways of estimating μ*R* for a given sample, and describe a software package for rapidly obtaining and applying the correction.

## 4 Validation Experiments

To explore the effects of the correction on real data, powder X-ray diffraction patterns were collected for four samples with varying absorption cross sections. To cover a large range of μ*R*, ZrO_2_, CeO_2_, and HfO_2_ were packed in Kapton (polyimide tubes) with varying inner diameters (IDs). To get a sense of how the μ*R*’s are distributed, the theoretical values were computed based on each sample composition, mass density, and capillary diameter using the XrayDB database.^35^ The mass density was determined by measuring the mass of the packed powder and the length of the powder bed. The sample list, along with the corresponding information, is presented in Table 1, ranging from μ*R* = 0.4 to almost 6. For reference, a subset of the samples (ID = 1 mm) was also measured using synchrotron X-rays.

**Table 1:** List of Samples That Were Measured on the Bruker Lab diffractometers Equipped with a Mo *K*_*α*_ Source^a^ ^35^

sample	ID (mm)	density (g/cm^3^)	μ*R*
ZrO_2_	0.635	1.009	0.40
	0.813	0.856	0.43
	1.024	1.122	0.71
CeO_2_	0.635	1.706	2.11
	0.813	1.435	2.28
	1.024	1.457	2.91
HfO_2_	0.635	1.741	4.08
	0.813	1.963	5.90
	Wire^b^		

a The samples were packed in Kapton (polyimide) tubes with different inner diameters (IDs). The density was calculated based on the amount of powder packed in a given segment of the cylindrical Kapton tubes. μ*R*’s were calculated using the database from XrayDB.^35^

b Powder was rubbed on the outside of a glass wire covered in grease. The density and radius of the wire and powder are therefore unknown, and a theoretical μ*R* cannot be computed.

We describe here our protocol for sample preparation and estimation of the density. We first take a Kapton straw of a suitable length and block one end with clay. This is then weighed and the weight recorded (*m*_0_). Powder is then scooped up with the tube, which is then tapped on the table to seat it. This is repeated several times to get a powder bed length of a few centimeters (for lab measurements, we need a long bed). Then, the Kapton straw with the powder is weighed again (*m*_1_). The difference (*m*_1_–*m*_0_) gives the mass of the powder in the Kapton tube. Finally, in the examples in the paper, a piece of cotton wool was inserted into the open end of the tube and pushed down onto the powder with a metallic wire. The length of the powder is then measured, and the volume of powder is computed from π*R* ^2^ *l*, where *l* is the length of the powder column. The sample density, ρ_S_, is then computed in units of g/cm^3^. Though it is not needed in practice, for the sake of interest, we compute the packing fraction from $f_{p}=\frac{\rho_{S}}{\rho_{t}}$ where ρ_*t*_ is the theoretical density of the material computed from the mass of atoms in the unit cell divided by the unit-cell volume. The packing fractions we got for the samples in this study were in the range 15% < *f*_p_ < 24% expressed as percentages.

The laboratory PDF measurements were performed on a Bruker D8 Discovery diffractometer equipped with a Mo *K*_α_ source (*K*_α_1_α_2__ double emission, average wavelength λ = 0.71073 Å) using capillary geometry to ensure a constant sample illumination. The configuration included a focusing Goebel mirror, a divergence slit of 1.0 mm for IDs 0.635 and 0.813 mm, and 1.2 mm for IDs 1.024 mm, a 2.5° axial Soller slit, a scattering guard after the source, and an additional 2.5° axial Soller slit in the diffraction beam before the detector. X-ray generator settings of 50 kV and 50 mA were employed. The acquisitions were conducted using the staircase-counting-time (SCT) measurement strategy described in our previous work that ensures increased counting statistics in the high-*Q* region.^4^

The SCT acquisition protocol consisted of 5 scans with a constant step size of 0.025°, decreasing the 2θ-range and increasing the counting time per step as shown in Table 2.

**Table 2:** Staircase-Counting-Time (SCT) Protocol for the Lab PDF Measurements on a Bruker D8 Discover Diffractometer

	2θ start	2θ stop	counting time (sec)
scan 1	2	140	1.8
scan 2	75	140	3.6
scan 3	107	140	7.2
scan 4	124	140	14.4
scan 5	132	140	28.8

Synchrotron total scattering measurements were conducted at ID31 at the European Synchrotron Radiation Facility (ESRF) in Grenoble, France. The sample powders were loaded into cylindrical slots (1 mm thickness) held between Kapton windows in a high-throughput sample holder. Each sample was measured in transmission with an incident X-ray energy of 75.00 keV (λ = 0.1653 Å). A Pilatus CdTe 2 M detector (1679 × 1475 pixels, 172 μm × 172 μm each) was positioned with the incident beam in the corner of the detector. The sample-to-detector distance was approximately 0.3 m. Background measurements for the empty windows were measured and subtracted. NIST SRM 660b (LaB6) was used for geometry calibration performed with the software pyFAI,^36^ followed by image integration, including a flat-field, geometry, solid-angle, and polarization corrections. Raw XRD data from each SCT scan were normalized in counts/second (CPS), accumulated into one data set, and then exported as a .xy file using the Bruker DIFFRAC.EVA software. A correction was then applied for beam polarization by dividing the laboratory data by $\frac{1+\cos^{2}\left(2\theta \right)}{2}$ , assuming the incident radiation is approximately unpolarized.^34^ From here onward, “uncorrected” data refer to polarization-corrected data, whereas “raw” data denotes the original measurements prior to polarization correction, to avoid any ambiguity. Data processing to obtain the PDF, after any preprocessing to correct for absorption, was done using PDFgetX3.^18^ The Fourier transform from *F*(*Q*) to *G*(*r*) was done with the parameters *Q*_max_ = 16.6 and 30 Å^–1^ for the Mo *K*_α_ and synchrotron data, respectively, *Q*_min_ = 1.0 Å^–1^, and *rpoly* in the range of 1.0–1.8. The value of *rpoly* was set according to normal protocols to ensure that the shape of the *F*(*Q*) function had a concave baseline while keeping *rpoly* less than the nearest neighbor bond length in the material.

Models were fit to the PDF data using Diffpy-CMI.^37^ As we did in our previous work,^4^ we fit models to uncorrected, corrected, and synchrotron data to evaluate the effect of the absorption correction by comparison to the fitted synchrotron data. Structural models for m-ZrO2, c-CeO_2_, and m-HfO_2_ were taken from the Inorganic Crystal Structure Database (ICSD) (entry IDs 80047, 184584, and 60902, respectively).^38–40^ The fit was performed on the Nyquist–Shannon (NS) grid to facilitate propagation of valid estimated uncertainties.^41^ The refined parameters include scaling variable *s*_1_, damping factor *Q*_damp_, broadening parameter *Q*_broad_, correlated motion parameter δ_2_, lattice parameters, and atomic displacement parameters (ADPs). The ADP constraints and fitted *r* range vary slightly for different data sets and will be presented separately for each data set.

## 5 Results

### 5.1 Assessment of the Absorption Correction for Different μ*R*’s

In Figure 2, by way of example, we show curves of A* for various choices of μ*R*. In Figure 2(a), μ*R* varies from 3.07 (lower dark blue curve) to 35.76. The μ*R* values are representative of samples of TiO_2_, ZrO_2_, SnO_2_, CeO_2_, and HfO_2_, respectively, measured with Mo *K*_α_ radiation in a 1 mm tube but are only chosen as representative of real materials from different rows in the periodic table for comparison.

**Figure 2:**
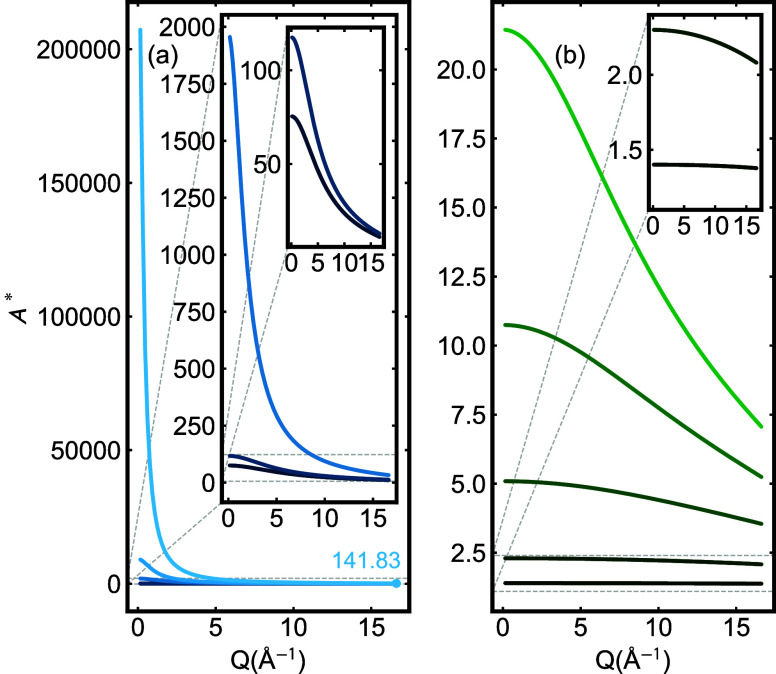
Absorption correction A* calculated using the brute force method for various values of μ*R*, over the entire *Q*-range for Mo *K*_α_ radiation. From bottom to top in (a) A* for μ*R* = 3.07 (dark blue), 3.53, 8.58, 14.08, 35.76 (light blue), and (b) A* for μ*R* = 0.2 (dark green), 0.5, 1, 1.5, and 2 (light green). The insets show the A* curves for lower μ*R*’s on expanded scales. In panel (a), the A* value for the largest *Q* at the highest μ*R* is labeled in the same light blue as the corresponding curve.

All of the curves fall from a high value at low-*Q* to a smaller value at high-*Q*. For the heavier elements, the low-*Q* signal would be multiplied by up to 200,000 times, falling to around 140× at the highest-*Q* of 16.5 Å^–1^. Experiments are obviously not tenable with a 10^5^ attenuation in signal, and for heavier elements at modest X-ray energies, thinner samples are needed, as is widely known. We explore this in more quantitative detail below.

In the smallest inset of Figure 2(a), we show the curves for more experimentally reasonable, albeit somewhat high, μ*R* values of around 3. In Figure 2(b), we show curves for μ*R* less than 3 (0.2 < μ*R* < 2). Even for these experimentally more realistic cases, the value of A* has a strong *Q*-dependence. For the higher range of μ*R*, the value of A* can change by ≈50% from the low-*Q* to the high-*Q* end. A μ*R* in the vicinity of unity is generally considered optimal (shown in the inset to Figure 2(b)). Below, we explore data quality for higher μ*R* values for the cases where it is difficult to make samples for PDFs that are sufficiently thin.

### 5.2 Exploration of Different μ*R* Cases

To understand how the A* curves vary in shape with μ*R*, we compute them and plot them scaled to go from zero to one, in Figure 3. They are normalized to go from zero to one, as we are interested in the shape of the curve and not its absolute magnitude in this analysis. The figure shows curves in the range from 0.05 ≤ μ*R* ≤ 45. All of the curves fall off more slowly, followed by a rapid falloff with increasing *Q* and finally a long tail in the high-*Q* region. What is characteristic of increasing μ*R* is that the crossover happens at a lower angle, and therefore lower *Q*. For the lower μ*R* values, the curves are relatively flat over a much wider angular range. The flatness of these curves for small μ*R* allows the absorption correction to be neglected in the *ad hoc* PDFgetX3 algorithm^18^ for the case of high-energy synchrotron X-ray measurements in the RAPDF^1^ geometry. In that geometry, the maximum 2θ angle is around 40–50°, much less than the 2θ_max_ = 140° in typical laboratory PDF measurements, which places the absorption correction even more in a flat region of the A* curves. For lower-energy X-rays, which are more absorbing and require wider measurement angles, whether measured on laboratory instruments or at the synchrotron, this absorption correction should be considered in most cases.

**Figure 3:**
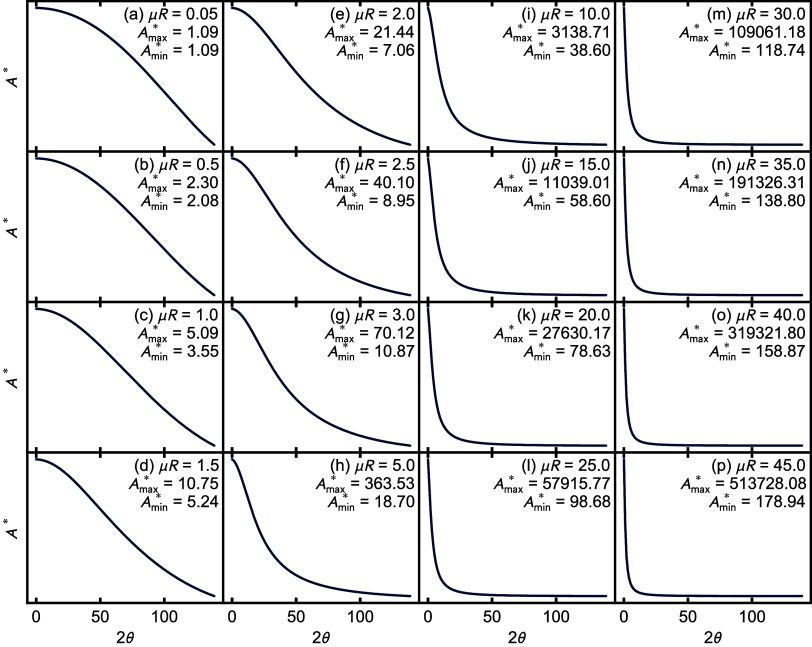
Comparison of the shape of absorption correction A* curves for the range of μ*R* values from 0.05 to 45. The A* curves are normalized from 0 to 1 to emphasize the shape of the curves vs 2θ for different values of μ*R*. The values of *A*_max_^*^ and *A*_min_^*^ reported in the panels are the maximal and minimal values, respectively, of A* before normalization, preserved to two decimal places, for the angular range shown (0 < 2θ < 140°).

The maximal and minimal absolute values of A* before normalization are also reproduced in each panel of the figure to give an idea about how large an angle dependence is required on an absolute scale. For μ*R* ≤ 1, even for the wide angular range of the data considered here, the angle dependence of the correction is quite small, but it grows rapidly for larger μ*R* values. By a μ*R* = 3, the low-*Q* correction is 7× larger than the high-*Q* correction, and there is a significant angular dependence, though the data are probably still usable after applying the correction we lay out here.

To summarize, scattering properties are optimal, and absorption corrections are minimal for μ*R* values of around 0.5. However, samples with μ*R* up to around 3 result in reasonable absorption corrections but should have a correction applied for data collection over a wide angle. For samples with larger μ*R* values, the corrections become large, and the data are not likely to be good.

### 5.3 Estimating μ*R* for a Sample

In this section, we consider a number of different ways for estimating μ*R* for a sample. At first sight, this is straightforward since this quantity can, in principle, be calculated from mostly known quantities. However, in practice, some quantities such as the sample density or its chemical composition, are often not well-known. Also, the model we used to compute the correction makes some assumptions that are not necessarily true in practice. For example, that of a parallel beam of the same width as the sample is not necessarily true in practice. We therefore seek to determine an “effective” μ*R*, μ*R*_e_, for our sample, which is the μ*R* that gives the most appropriate A* curve given our experimental conditions. We compare a number of different approaches for determining μ*R*_e_ values by evaluating their effect on the refined structural parameters. We would like to understand empirically which approaches for estimating μ*R* are preferred, as well as, in general, how big an effect the A* correction has on refined parameters.

We first consider a number of different ways for estimating μ*R* for a sample. Later, we compared them by modeling. The μ*R* of a sample can be measured directly for a given diffractometer setup by measuring the X-ray attenuation of the capillary specimens placed in the incident beam, as shown in Figure 4. In this measurement, the sample stage is moved vertically (defined here as the *z*-axis), traversing the incident beam. In our case, for this, the Bruker Lynxeye detector is set to 0D-mode, which means that the whole detected area is integrated.

**Figure 4:**
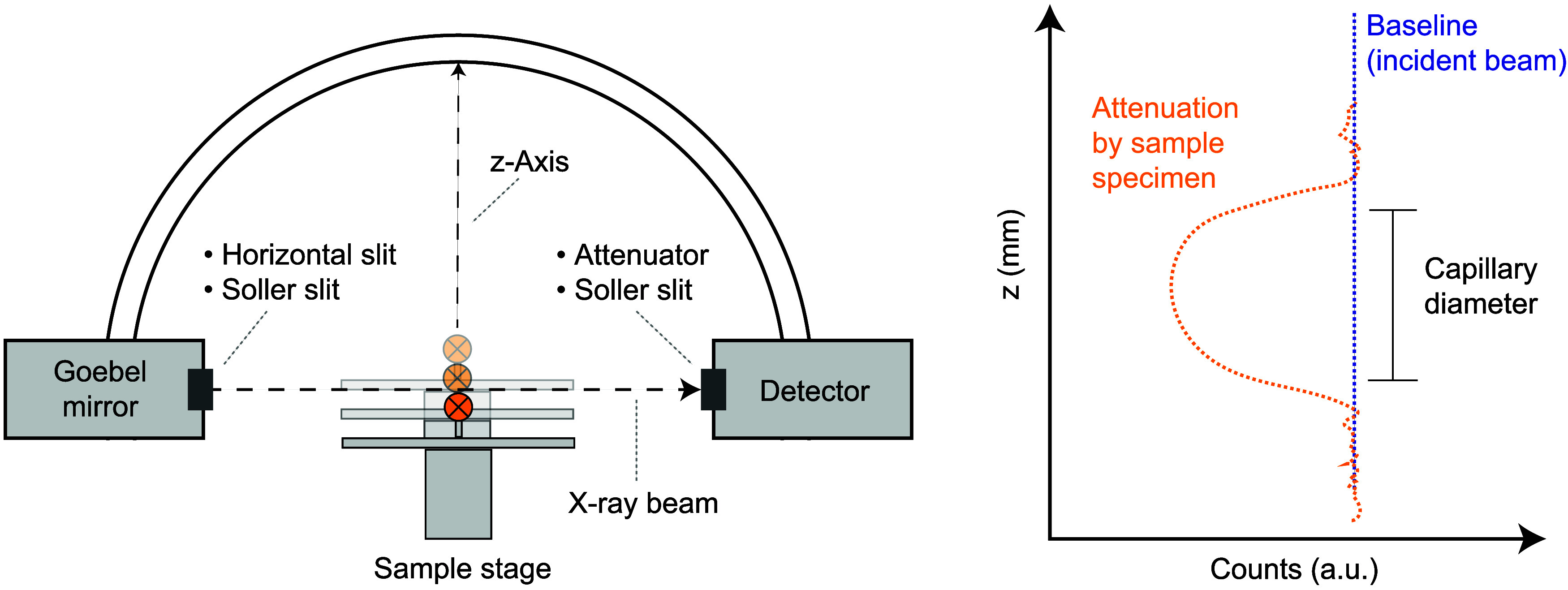
Scheme of the diffractometer setup for direct measurements of the sample μ*R*, called here *z*-scans. The sample stage is moved along *z* so the sample traverses the incident beam while recording the X-ray intensity as a function of *z*. The capillary sample is displayed as an orange circle with its principal axis perpendicular to the viewing plane. This results in a U-shaped profile (orange curve on the right). The capillary diameter, marked by the black bar on the right, is slightly shorter than the full opening of the orange U-shaped curve because of the finite width of the beam.

The resulting absorption profile can be fit with a function that is a convolution of the width of the incident beam and the absorption characteristics of the sample, assuming it is cylindrical, as we describe below.

We tested absorption scans where the incident beam height was made narrower and more tightly collimated than was the case for the actual measurement of the PDF data. We also tested making the absorption scan with wider incident beam heights, including the height used in the actual PDF measurements. To accomplish the experiment with the narrower beam, either the active channels on the detector were reduced or a horizontal slit was placed in the incident beam before the sample to narrow the beam. The former limits the number of pixels that are active, limiting the active area on the detector to a horizontal stripe. A single channel is a horizontal strip, 0.075 mm in height, with an active area on the detector. Here, we use the terms “reduced” and “open” channel conditions to refer to cases where 3 and 191 active channels are kept open, respectively. The latter masks the beam and limits the beam divergence. We found that both have a similar effect on the resulting U-shaped *z*-scan. We then compared three methods to determine μ*R* from the *z*-scans.

*Method 1:* An approximate μ*R* is determined using

8 $$\mu R_{m1}=\frac{1}{2}\ln\left(I_{\max}/I_{\min}\right)$$

where *I*_max_ ≈ *I*_0_ and *I*_min_ ≈ *I*_0_·e^–2μ*R*^. The rationale is that the minimum attenuation (*I*_min_) is reached when the center of the capillary aligns with the center of the X-ray beam. If the vertical beam height is small enough relative to the capillary diameter, then at this point, the sample thickness does not change much across the beam diameter, and the measurement resembles a standard μ*t* measurement of a sample of uniform thickness *t*. Knowing μ*t* at the position of the diameter, and the diameter, we can obtain μ and, therefore, μ*R* for the capillary. This reasoning breaks down when the beam height becomes comparable to the sample height.

*Method 2*: We carry out a fit of the *z*-scan curve to a model that assumes a circular cross-section capillary of uniform density and a parallel X-ray beam of height *h*, which estimates μ*R*. The mathematical details are included in Supporting Information, Section S4. The fit yields μ*R*_*m*2_ by fitting parameters μ, *D*, *h*, *I*_0_, *z*_0_, the height of the center of the capillary, and *m*, a linear coefficient for *I*_0_ that we found depends on *z*. This model ignores the effects of the sample container, which we expect to have a negligible effect for thin polyimide or quartz tubes at Mo or Ag *K*_α_ energies. It will be a less good approximation if the sample container absorption is significant.

*Method 3:* Method 3 is the same as Method 2 except that for μ*R*_*m*3_, the capillary diameter, *D*, is fixed to the known value from the manufacturer and not allowed to vary, as it is in Method 2. In an ideal world, Method 2 would return a fit diameter that is very close to the known physical diameter, but we found that this was not always the case due to inadequacies in our model, and we wanted to understand the effect this has on the results.

It is also possible to estimate μ*R* theoretically using the known X-ray wavelength, sample composition, and densities and/or powder packing fractions, and so we define also *Method 4:* This makes use of tabulated attenuation coefficients from online resources such as XrayDB,^35^ APS,^42^ and the NIST Standard Reference Database 126.^43^ Here, we calculate μ*R*_th_ values using a measured mass density for our loaded samples and the lookup tables that makes use of the python XrayDB database.^35^ The computing details are described in Supporting Information, Section S5.

For convenience, we have developed μ*R* calculators in the Python software package diffpy.utils^44^ for Methods 2 and 4, which are free to use. For Methods 2 and 3, we also explored various experimental settings for the *z*-scan, including varying X-ray beam heights *h* and detector channel conditions. The full set of μ*R* values and fitted parameters that we tried is presented in Tables S13 and S14 in the Supporting Information. Here, we confine ourselves to *z*-scan data for CeO_2_ with ID = 0.635 mm, which was chosen since it had a high, but not excessive, μ*R* (μ*R*_th_ = 2.11), which, according to the μ*R* curves in Figure 3, suggests that an inappropriate estimation of this will result in a significant effect on the measured intensities. Examples of measured *z*-scans are shown in Figure 5 as blue circles, computed using Method 2, with fits shown as red lines. The theoretical curve for the sample transmission before convolution with the beam height is shown as brown curves.

**Figure 5:**
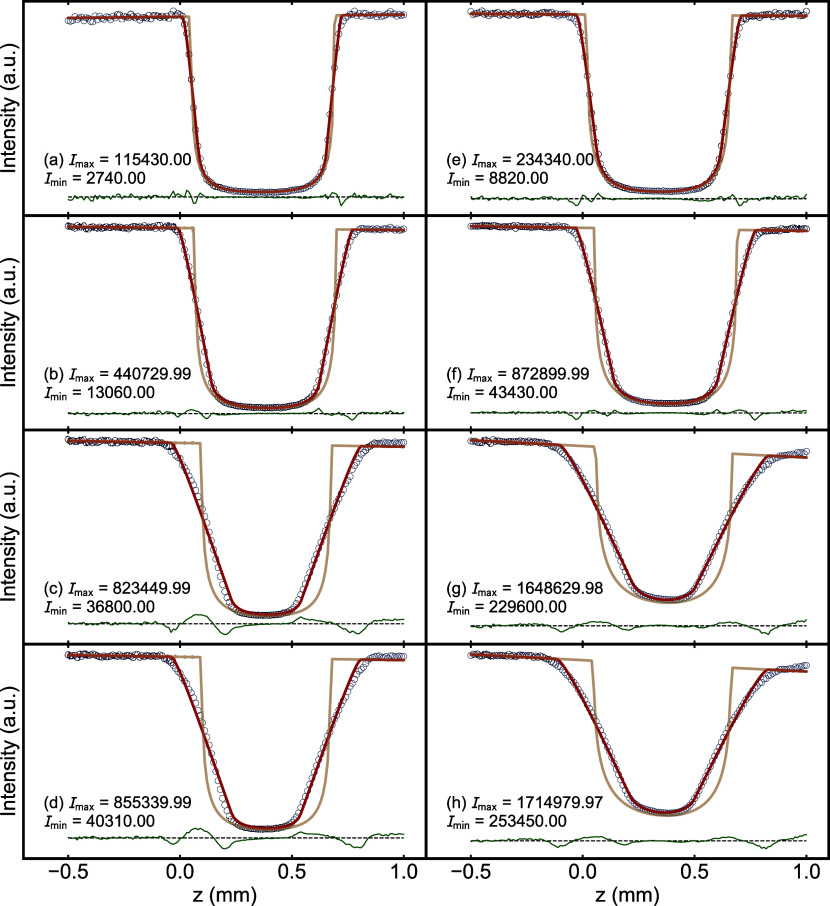
Examples of *z*-scans computed using Method 2 (blue circles) with model fits (red lines) for CeO_2_ with an ID of 0.635 mm. The green lines offset below show the residuals. In each case, the brown U-shaped curve is the unconvoluted intensity, which gives an indication of the edges of the capillary. The panels are arranged, so side-by-side panels are measured with reduced (left) and open (right) detector channel settings and measured with increasing height of beam slit going down the columns *h* = 0.05, 0.2, 0.6, and 1 mm.

When comparing across rows where *h* is constant, we observe that the maximal intensity *I*_max_ is around twice as large for open channels, while the minimal intensity *I*_min_ is about 4 times larger. This results in lower μ*R*_*m*2_’s for open detector channels. In addition, as *h* increases (comparing the down columns), we find that the slope of the U-shape curve (blue curve) becomes less steep. In all cases, the fits of our model are satisfactory, albeit returning quite different μ*R*_*m*2_’s.

The different μ*R*_*m*2_’s are summarized in Figure 6(a,b), along with those of μ*R*_*m*1_ and μ*R*_*m*3_, for the same experimental conditions considered in Figure 5.

**Figure 6:**
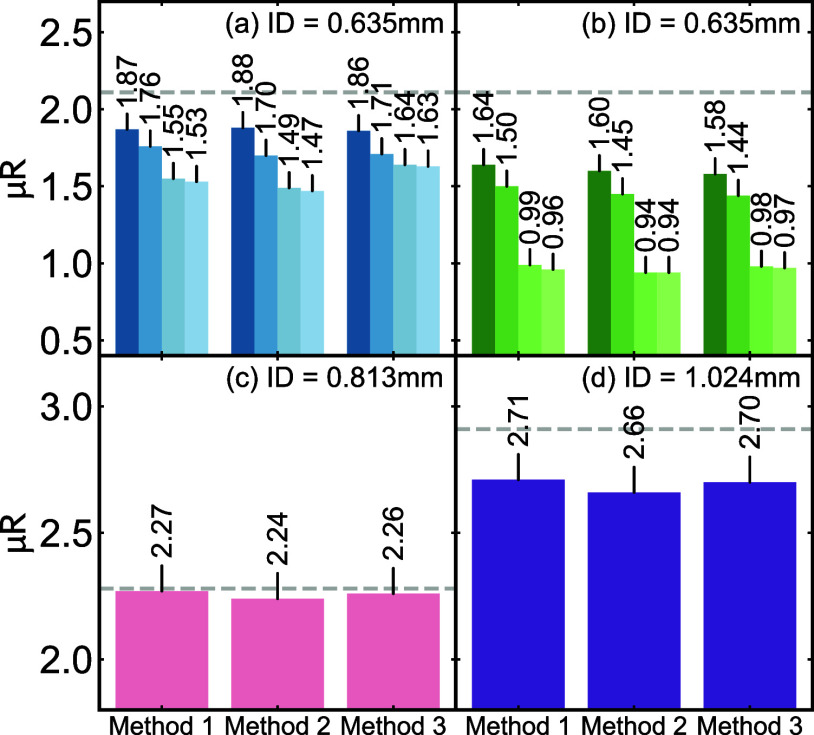
μ*R* values for different CeO_2_ data sets determined from *z*-scans using varying incident beam heights (*h*) and channel conditions. In all panels, the IDs are reported with μ*R*_*m*1_, μ*R*_*m*2_, μ*R*_*m*3_, and μ*R*_th_ shown. μ*R*_th_ for panels (a–d) are 2.11, 2.11, 2.28, and 2.91, respectively, as indicated by the dashed gray line. In panels (a, b), lighter color bars indicate larger *h* values, *h* = 0.05, 0.2, 0.6, and 1 mm. Panel (a) shows the reduced channel detector condition, while PANEL (b) shows the open-channel detector condition. Panels (c, d) show data with reduced channels with *h* = 0.1 mm.

Comparing across the methods, we see that for each experimental setting indicated by the same color, Methods 1–3 give very similar values. This means that we could use any of these methods to estimate μ*R*, and it would not matter, and so hereafter, we only report values for Method 2. On the other hand, Method 4, using the theoretically determined μ*R*, indicated by the dashed gray line, gives a consistently higher value.

However, we see large variations in the estimate of μ*R* depending on the experimental conditions used to measure it. Comparing across color lightness (beam height, *h*) and between panels (a) and (b) (reduced vs open detector channels), the resulting μ*R* can vary by almost a factor of 2.

A question, therefore, arises: which of these values of μ*R* is the most appropriate for correcting the XRD data for PDF analysis, and that is explored in the next Section. Here, we can make some observations that help us understand why we see these variations.

We first note that the beam height, limited by physical slits upstream of the sample and the channel settings of the detector, has a similar effect. The former limits the effective area of the sample that is illuminated, and the latter limits the area of the beam on the detector for which it is accounted for. Comparing panels (a) and (b), we always get slightly lower μ*R* values with the open-channel setting, but the difference is small when *h* is small (i.e., 0.05 and 0.2 mm).

We note that another important length-scale in the problem is the diameter of the capillary (0.635 mm in this case). When *h* is much smaller than the capillary diameter, we get larger estimates of μ*R* that are also closer to the theoretical μ*R*’s but much smaller estimates of μ*R* when the *z*-scan is measured with beam-slit heights comparable to or larger than the sample capillary. To remove uncertainty for experimenters, one approach is to recommend that the PDF data are collected with a beam height that is approximately the same as the capillary diameter.

### 5.4 Understanding the Effect of Different μ*R* Correction Magnitudes on Fits and Refined Parameters

We next investigate the effect on the fit quality and refined parameters of correcting the ceria PDFs using different μ*R*’s. We use CeO_2_ data from the 0.635 mm capillary as an example, where the absorption effect is moderately strong. For the purpose of comparison, in addition to the uncorrected data, we consider three different μ*R* values for the corrected data: a small μ*R* = 0.97 that is close to the smallest experimental value, μ*R* = 1.53, which is a middle experimental value, and a purposefully overestimated μ*R* = 5. We do this by computing structure functions and PDFs from the measured data after each of the three corrections and fitting the PDFs of the ceria crystal structure model using Diffpy-CMI.

The effects of these corrections on the raw data, *F*(*Q*), and *G*(*r*) are shown in Figure 7. We consider the effect on the high-*Q* region of the raw data (Figure 7(b–d)). In these plots, the curves are normalized, so the strongest peak in each curve is set to unity, allowing us to conveniently compare the behavior at high-*Q*. Similar to what was shown in Schertenlieb et al.,^4^ albeit in that case for lower absorbing samples, we see that the absorption correction reduces an overall rise in the data while at the same time suppressing the high-*Q* signal. In other words, neglecting the correction will result in an amplified signal at high-*Q*, which might resemble an underestimated Debye–Waller factor in subsequent refinements. Unsurprisingly, the effect on the signal becomes larger as the μ*R* correction is increased. We note that the polarization correction does not remove the rise at high-*Q*, but the additional absorption corrected does remove it almost completely with a μ*R* = 1.53 (comparing light and dark blue curves in Figure 7(c)). For the overestimated μ*R* = 5, which we believe to be a significant overestimate of the actual sample absorption, the signal almost has a small decrease, similar to synchrotron data.^4^

**Figure 7:**
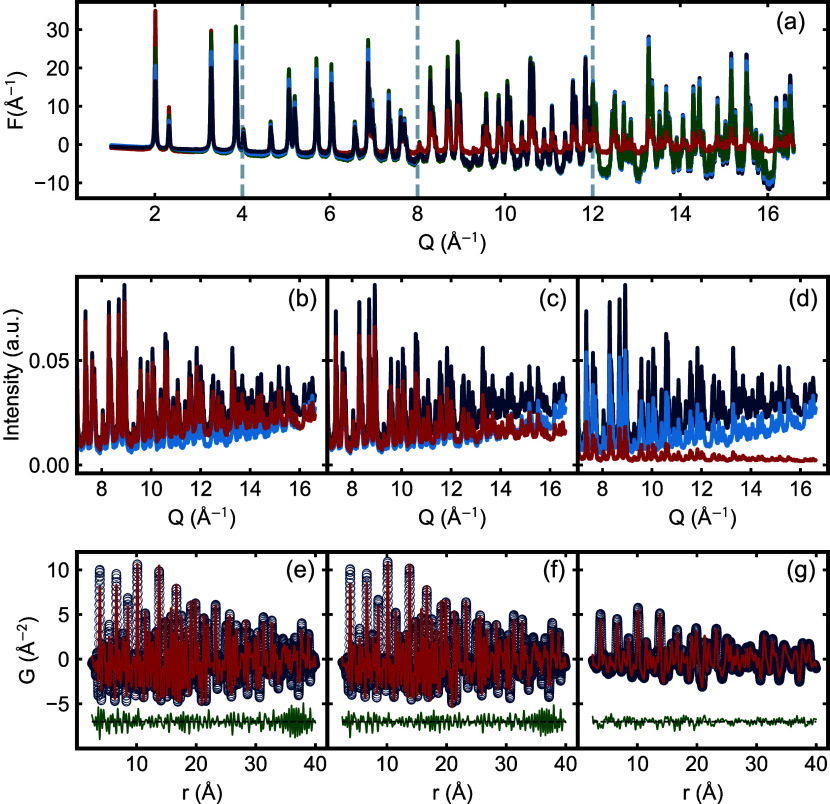
Comparison of CeO_2_ data (ID = 0.635 mm) with and without absorption correction, using small (μ*R* = 0.97), middle (μ*R* = 1.53), and overestimated (*μR* = 5) values. Panel (a) shows *F*(*Q*), where the dark blue, light blue, green, and red curves represent no correction and corrections with small, middle, and overestimated μ*R*, respectively. The curves overlap or change in amplitude across *Q*-ranges, marked by the light gray dashed vertical line, with the order of plotting adjusted to reflect changes without disrupting overall trends. Panels (b–d) show the raw (light blue), polarization-corrected intensity (dark blue), and fully corrected intensity (red), scaled so that the highest intensity peak equals to one. Plots are shown on a *Q* scale, focusing on *Q* > 7 Å^–1^. Panels (e–g) show the best-fit calculated PDFs (red lines) on top of their respective measured, absorption-corrected PDFs (blue circles). The PDFs were fitted between *r*_min_ = 1.0 and *r*_max_ = 40.0. The corrections in THE left column (b, e) correspond to the small μ*R*, the middle column (c, f) to the middle μ*R*, and the right column (d, g) to the overestimated μ*R*.

The effect on *F*(*Q*) plotted over the whole range of *Q* is shown in Figure 7(a), where we find underestimation in the low-*Q* signal and overestimation in the high-*Q* signal when comparing uncorrected to corrected data. The progressive suppression of the high-*Q* signal (*Q* > 12 Å^–1^) with increasing correction is very clear.

We turn to fits of structural models to the PDF to understand the effect of the corrections on the fit quality and refine structural parameters.

The *G*(*r*) fits for the corrected data are shown in Figure 7 (e–g). A larger version is shown in Figure S15 in the Supporting Information. All of the curves result in reasonable model fits, with small misfits, as evident in the green difference curves. The *R*_w_ value for the uncorrected case is approximately 0.49, and it improves to 0.43, 0.36, and 0.27 for small, middle, and overestimated μ*R*, respectively. The fit quality, as measured by the *R*_w_, and visually in the difference curve, gets better with increasing magnitude of the absorption correction. This trend occurs even when we use a correction that is known to be too strong. This means that fit quality alone, as measured by *R*_w_, is not a good measure of the best absorption correction to use. Of course, the purpose of the fits is to obtain high-quality values for refined structural parameters. We therefore turn to an analysis of the refined parameter values obtained with different degrees of absorption correction.

The refinement results for the three selected μ*R* corrections are shown in Table 3. We are most concerned about the refined values of structural parameters that are, in this case, *a*, Ce­(*U*_iso_), and O­(*U*_iso_). The effect of the correction on the lattice parameter is small, varying at the thousandths of an Ånsgröm level, but does show a small decrease in lattice parameter with increasing strength of correction. The Ce­(*U*_iso_) and O­(*U*_iso_) values vary with the μ*R*. Ce­(*U*_iso_) increases with increasing absorption correction in line with the progressive suppression of the high-*Q* signal by the correction. On the other hand, O­(*U*_iso_) decreases with increasing correction magnitude, and then increases again for the overcorrected data, but this may reflect the fact that the oxygen contributions to the measured PDFs in CeO_2_ are very small, and so these ADPs are not very reliable.

**Table 3:** Results of the Refinement for CeO_2_ Data (ID = 0.635 mm) for Synchrotron, Uncorrected, and Corrected Data with Selected μ*R*’s, Over *r*_min_ = 1.0 and *r*_max_ = 40.0^a^

parameter	synchrotron	uncorrected	μ*R* = 0.97	μ*R* = 1.53	μ*R* = 5
*s*_1_	0.37434(21)	0.3919(13)	0.4379(13)	0.4698(13)	0.3350(6)
*Q*_damp_	0.02386(4)	0.02831(20)	0.02787(20)	0.02700(19)	0.01630(19)
*Q*_broad_	0.01814(7)	0.0246(4)	0.0243(4)	0.02415(34)	0.02407(20)
δ_2_	9.07(4)	12.962(35)	13.322(21)	13.589(14)	14.4085(16)
*a*	5.414232(7)	5.40344(5)	5.40313(4)	5.40286(4)	5.40219(4)
Ce(*U*_iso_)	0.003571(5)	0.003028(24)	0.003323(24)	0.003725(26)	0.00932(4)
O(*U*_iso_)	0.04120(10)	0.0854(13)	0.0783(11)	0.0717(9)	0.0704(5)
*Q*_max_	30.0	16.6	16.6	16.6	16.6
grid	0.10472	0.189253	0.189253	0.189253	0.189253
*R*_w_	0.160823	0.492458	0.428941	0.355725	0.26695
χ_red_^2^	961.528958	667.00816	525.159995	390.974207	533.070925

a The μR’s are the values used for each correction.

To decide which μ*R* values are optimal, we compare to fits to the synchrotron data. The refined lattice parameters from the lab data are closer to reported values,^39^ indicating that the synchrotron data overestimates the lattice parameter, which may be related to a calibration issue with the synchrotron data. Among the three selected correction strengths, we find that the middle μ*R* = 1.53 provides *U*_iso_ values that agree the best with the synchrotron data.

A detailed exploration of μ*R*_e_ for all CeO_2_ data is presented in Supporting Information Section S6, where we propose a recommended reference value. Additional examples for both low and high sample absorption cases are discussed in Supporting Information Section S7.

## 6 diffpy.labpdfproc Software for Absorption Corrections

The corrections described here have been implemented in an easy-to-use Python package, diffpy.labpdfproc, as part of the diffpy^45^ family of software packages. It can be used to compute absorption corrections and apply them to measured data before the data are fed into, for example, PDFgetX3, to obtain PDFs.

The software can be easily installed from the Python Package Index (Pypi)^46^ or from conda-forge^47^ and so is straightforward to use alongside other diffpy programs. Full instructions for installation and use can be found at the GitHub readme^48^ and the online documentation.^49^

To use this tool, you only need your 1D diffraction pattern data along with either the μ*R* value, a *z*-scan file, or relevant chemical information. The program is fast and easy to use. By default, we use a fast calculation for the A* correction, described in Supporting Information Section S3. It can be run on a single file or on directories of files. It also implements the more computationally intensive brute force calculation method described in Section 3. diffpy.labpdfproc can also be used to facilitate estimating μ*R* for a sample of known composition before making the samples part of the experiment design.

## 7 Recommended Protocols for Sample Preparation and Absorption Correction for PDF Measurements in Laboratory Settings

Here, we provide our recommended protocols for handling sample absorption in detail. For other experimental decisions for PDF experiments from lab sources, please refer to our previous work.^4^

Recommendations for sample preparation:

1. Ideally, select a capillary or Kapton tube with an inner diameter that will result in a μ*R* in the range 0.5–1.
2. If, for some reason, this is difficult, a μ*R* up to 3 is acceptable, and up to 4 may be workable. Above this is unlikely to give acceptable results.
3. For highly absorbing samples, it is possible to prepare the sample by smearing light grease (e.g., Vaseline) on the outside of a thin glass fiber and rolling it in the powder ground as finely as possible.

Recommendations for data corrections:

1. If it is not done by the instrument software, apply a polarization correction to your data using diffpy.labpdfproc.
2. If possible, obtain a measured μ*R* for the sample using the *z*-scan method in diffpy.labpdfproc with a small effective beam height, either by masking the beam with a slit or by decreasing the active area on the detector. If a *z*-scan is not possible, you can obtain a μ*R* theoretically but using a measured mass density and composition and using diffpy.labpdfproc.
3. If a *z*-scan is not possible and the density cannot be measured, estimate μ*R* using the composition of the sample and the theoretical density. This may be obtained with the help of diffpy.labpdfproc. You will need to estimate a packing fraction. We recommend, as a default, a value of 0.25. A value of 0.5 is often mentioned in the literature, but we found that samples made of compacted loose powder rarely get to this value and, as mentioned above, even then result in an overestimate, which is why we recommend the lower value of *f*_p_ = 0.25 as a reasonable guess.
4. For small theoretical μ*R* values (≤2.2), the optimal μ*R* can be obtained from *z*-scan measurements using the actual experimental settings but with reduced channels, while for larger μ*R*’s, it can be approximated by multiplying the theoretical value by around 1.3–2×.

## 8 Conclusion

In contrast to rapid acquisition PDF experiments,^1^ PDF experiments carried out on laboratory diffractometers, or at synchrotrons but with low X-ray energy, require absorption corrections. Here, we revisit the nature of absorption corrections and their effect on the resulting structural refinements for laboratory PDF measurements, which are becoming more popular. We report a new software program, diffpy.labpdfproc, that can carry out absorption corrections in a fast and straightforward way before data are then propagated to the popular PDFgetX3 program. We also assessed the effects of different absorption corrections on subsequent PDF refinements as well as explored different methods for determining the best μ*R* values in a particular experimental situation. The goal is to make this step as straightforward as possible so that it can be easily incorporated into laboratory PDF experimental workflows. As a result, we present protocols for sample preparation to improve data quality.

## Supplementary Material



## Data Availability

Data used for all of the plots in the manuscript are available on Zenodo at 10.5281/zenodo.16900908.

## Notes

Derivation of the path length of an X-ray through the sample; derivation of the result that *A** depends on the product μ*R*; fast calculation of the absorption correction using a polynomial approach; estimation of μ*R* using model fitting of *z*-scans; estimation of μ*R* using a theoretical database; selecting optimal μ*R* in different situations; and refinement across extended μ*R* values (PDF)
