# Supplementary material for: Absorption Correction for Reliable Pair Distribution Functions from Low Energy X‑ray Sources
Source: Cryst Growth Des. 2026 Jan 21;26(3):1036–47. doi: 10.1021/acs.cgd.5c00551 (PMC12879541; doi:10.1021/acs.cgd.5c00551)
Supplement: Supplementary file 1 [file cg5c00551_si_001.pdf]

Supporting Information for:

An Absorption Correction for Reliable Pair-Distribution Functions from Low Energy X-ray Sources

Yucong Chen,[†,¶] Till Schertenleib,[‡,¶] Andrew Yang,[†] Pascal Schouwink,[‡] Wendy L. Queen,[‡] and Simon J. L. Billinge[*,†]

†*Department of Applied Physics and Applied Mathematics, Columbia University, New York, NY 10027, USA*

‡*Institute of Chemical Sciences and Engineering (ISIC), École Polytechnique Fédérale de Lausanne (EPFL), Sion, CH-1951, Switzerland*

¶*Y.C. and T.S. contributed equally to this work*

E-mail: sb2896@columbia.edu

S1 Derivation of the path length of an x-ray through the sample

We derive here the relations for determining $\ell_v(2\theta)$, the path length through the sample of an x-ray scattered at angle 2θ from the vth voxel. As shown in Eq. 3 in the main text, this is given by the sum of the path lengths of the incoming and outgoing x-ray paths, ℓ_v^i and ℓ_v^o, respectively.

In our simplified model we assume that the incoming beam is parallel (non-diverging). The geometry is shown schematically in Fig. 1 of the main paper, where the circle in this figure represents the cross-section of the capillary and the incoming beam arrives horizontally from the left. Placing the origin of our coordinate system at the center of the circle we can consider a pixel, v, located at position with coordinates (x_v,y_v) (as examples, see the large grey dots in Fig. 1). The incident path length will be given by $\ell_v^i = |x_v - x_i|$, where the coordinates where the beam enters the capillary circle are (x_i, y_i) (indicated by the red dots in Fig. 1). Points on the capillary circle satisfy $x^2 + y^2 = R^2$, where $R = \frac{D}{2}$ is the radius of the capillary circle. Since $y_i = y_v$ which is known, we take the negative value and get that $x_i = -\sqrt{R^2 - y_v^2}$ and

$$\ell_v^i = \left| x_v + \sqrt{R^2 - y_v^2} \right| . \tag{1}$$

To get the path length of the out-going x-ray we notice that, if the coordinates of the point on the circle where the ray leaves the capillary are (x_o,y_o) (indicated by the yellow dots in Fig. 1), then

$$\ell_v^o(2\theta) = \sqrt{(x_o - x_v)^2 + (y_o - y_v)^2}. \tag{2}$$

Since x_v and y_v are known, it remains to find x_o and y_o.

When $2\theta \neq 90°$, we find the yellow line equation $y = ax + b$ that passes through both the voxel and the exit point, where its slope $a = \tan(2\theta)$ is determined by the scattering angle and its intersection point can be computed from the grid points $b = y_v - x_v \cdot \tan(2\theta)$. Therefore, we have that $x_o = \frac{y_o - b}{a}$. Since the exit point is an intersection point that passes through the line and the circle, we solve by establishing simultaneous equations for the circle

and the line. We then have:

$$x_o^2 + y_o^2 = R^2 \tag{3}$$

$$\left(\frac{y_o - b}{a}\right)^2 + y_o^2 = R^2 \tag{4}$$

$$(1 + a^2)y_o^2 - 2by_o + (b^2 - a^2R^2) = 0. \tag{5}$$

Since $a^2 \geq 0$, $1 + a^2 \neq 0$, and we can use the quadratic formula to get y_o:

$$y_o = \frac{2b \pm \sqrt{4b^2 - 4(1 + a^2)(b^2 - a^2R^2)}}{2(1 + a^2)} \tag{6}$$

$$= \frac{b \pm a\sqrt{(1 + a^2)R^2 - b^2}}{1 + a^2}. \tag{7}$$

As we consider only voxels within the cylinder ($x_v^2 + y_v^2 \leq R^2$), the discriminant of this quadratic is positive:

$$(1 + a^2)R^2 - b^2 = R^2 + a^2R^2 - (y_v - ax_v)^2 \tag{8}$$

$$= (R^2 - y_v^2) + (a^2R^2 - a^2x_v^2) + 2ax_vy_v \tag{9}$$

$$\geq x_v^2 + a^2y_v^2 + 2ax_vy_v \tag{10}$$

$$= (x_v + ay_v)^2 \tag{11}$$

$$\geq 0. \tag{12}$$

Since we only have to consider paths leaving the circle in direction of the detector, the exit point is above the grid point thus is the upper intersection point. So we take the positive

value here. Substituting and rearranging the equations, we get:

$$x_o = \frac{\left(\sqrt{(1+\tan^2(2\theta))R^2 - (y_v - x_v\tan(2\theta))^2} - y_v\tan(2\theta) + x_v\tan^2(2\theta)\right)}{1+\tan^2(2\theta)} \tag{13}$$

$$y_o = \frac{\left(y_v - x_v\tan(2\theta) + \tan(2\theta)\sqrt{(1+\tan^2(2\theta))R^2 - (y_v - x_v\tan(2\theta))^2}\right)}{1+\tan^2(2\theta)}, \tag{14}$$

which can then be plugged back into Eq. 2.

This function is not well behaved in the vicinity of $2\theta = 90^\circ$. At $2\theta = 90^\circ$, $x_o = x_v$ and so $y_o = \sqrt{R^2 - x_v^2}$, and

$$\ell_v^o(2\theta = 90^\circ) = \left| y_v - \sqrt{R^2 - x_v^2} \right|. \tag{15}$$

S2 Derivation of the result that A^* depends on the product μR: Express $\ell_v(2\theta)$ in radius R

From our previous expressions for $\ell_v(2\theta)$, Eq. 1, Eq. 2, Eq. 15, we find that if we can express x_v and y_v linearly in terms of R, then we can factor R out for all $\ell_v(2\theta)$ and therefore use the product μR as an independent variable of A^* rather than μ and R separately.

Here, we present a method for expressing the Cartesian coordinates (x_V, y_V) of any point V inside a circle in terms of the radius R. This process is also visualized in Fig. S1 below. Consider the line segment OV from the origin, O, at the center of the circle to the point V inside the circle. Draw a line perpendicular to OV that goes through V. This line intersects the boundary of the circle at two points. Choosing one such point and denoting it as A, we establish the radius $OA = R$. Let $\alpha_V = \angle VOA$, then $\cos(\alpha_V) = OV/OA$, which gives us $OV = R\cos(\alpha_V)$. Transitioning to polar coordinates, we have that $x_V = OV\cos(\beta_V)$ and $y_V = OV\sin(\beta_V)$, where β_V represents the angle between the positive $x-$axis and OV. We therefore have $x_V = R\cos(\alpha_V)\cos(\beta_V)$ and $y_V = R\cos(\alpha_V)\sin(\beta_V)$.

Denote α_V as the angle formed between the line segment connecting the V'th pixel to

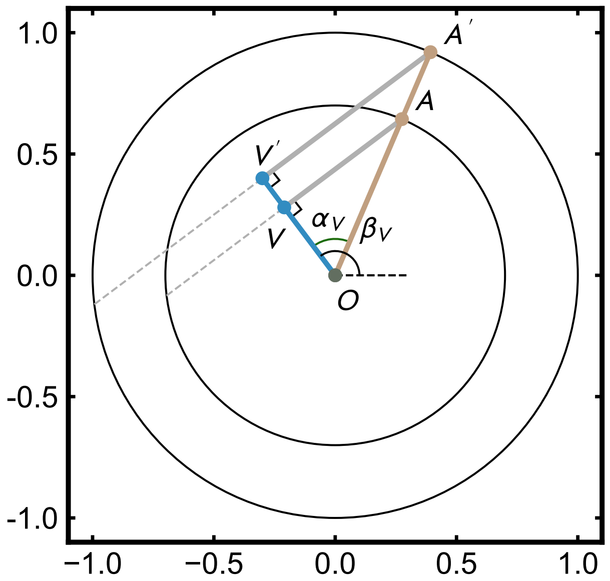

Figure S1: Visualization of the same V'th pixel for circles with varying diameters. The pixels are denoted by the blue dots, V and V', for the small and large circle, respectively. The blue lines represent the line segments connecting each pixel to the origin, and the dashed grey lines are the perpendicular lines intersecting each circle at two points. Here, we pick the light brown upper intersection point as A and A'. These light brown lines thus represent the radii. We can then find α_V and β_V, which are the same for V and V'.

the origin and the radius of the circle to the origin, and β_V as the angle between the positive x-axis and the line segment connecting the pixel to the origin. Since $x_V = R\cos(\alpha_V)\cos(\beta_V)$ and $y_V = R\cos(\alpha_V)\sin(\beta_V)$, we have the following:

$$\ell_V^i = \left| R\cos(\alpha_V)\cos(\beta_V) + \sqrt{R^2 - (R\cos(\alpha_V)\sin(\beta_V))^2} \right| \tag{16}$$

$$= \left| \cos(\alpha_V)\cos(\beta_V) + \sqrt{1 - \cos^2(\alpha_V)\sin^2(\beta_V)} \right| R, \tag{17}$$

$$\ell_V^o(2\theta = 90^\circ) = \left| R\cos(\alpha_V)\sin(\beta_V) - \sqrt{R^2 - (R\cos(\alpha_V)\cos(\beta_V))^2} \right| \tag{18}$$

$$= \left| \cos(\alpha_V)\sin(\beta_V) - \sqrt{1 - \cos^2(\alpha_V)\cos^2(\beta_V)} \right| R, \tag{19}$$

and

$$\ell_V^o(2\theta \neq 90^\circ) = \sqrt{(x_o - x_V)^2 + (y_o - y_V)^2} \tag{20}$$

$$= \sqrt{G^2 + H^2} R, \tag{21}$$

where

$$E = \sqrt{1 + \tan^2(2\theta) - \cos^2(\alpha_V)\big(\sin(\beta_V) - \cos(\beta_V)\tan(2\theta)\big)^2} \tag{22}$$

$$F = \cos(\alpha_V)\big(\sin(\beta_V) - \cos(\beta_V)\tan(2\theta)\big) \tag{23}$$

$$G = \frac{E - \tan(2\theta)F}{1 + \tan^2(2\theta)} - \cos(\alpha_V)\cos(\beta_V) \tag{24}$$

$$H = \frac{F + \tan(2\theta)E}{1 + \tan^2(2\theta)} - \cos(\alpha_V)\sin(\beta_V) \tag{25}$$

$$x_o = \frac{E - \tan(2\theta)F}{1 + \tan^2(2\theta)} R \tag{26}$$

$$y_o = \frac{F + \tan(2\theta)E}{1 + \tan^2(2\theta)} R. \tag{27}$$

Since α_V, β_V, and 2θ do not change for different R, we can factor out R from $\ell_V(2\theta)$. Denote $\ell_V(2\theta) = R \cdot d\ell_V(2\theta)$, using Eq. 7 in the main paper, we have

$$A^* = \frac{N_V}{\sum_V e^{-\mu R \cdot d\ell_V(2\theta)}}. \tag{28}$$

This concludes the proof that the same μR would give the same A^* and we can consider it as an independent variable.

S3 Fast calculation of the absorption correction using a polynomial approach

Part of the popularity of PDFgetX3 is the simplicity and speed of applying *ad hoc* corrections to the diffraction data that require little user input and still result in reliable PDFs.[1] For longer wavelength x-rays where absorption is important we would like a similarly quick and straightforward absorption correction that can be incorporated into the PDFgetX3 workflow. As mentioned in Section 2 in the main paper, many efficient computation methods have been proposed. For example, Dwiggins[2] developed an approach based on double integrals involving the hyperbolic function of cosh, achieving relative errors of $\leq 0.1\%$ for $0 < \mu R < 2.5$, although it suffers from a weak singularity. Ida[3] further analyzed this issue and compared Dwiggins' method with that of Thorkildsen & Larsen, which also uses a similar double integral approach involving the trigonometric function of sin. The latter approach was found to be more efficient and more accurate, reaching relative errors of $\leq 10^{-6}$. Another widely used approximation is the Lobanov & alte da Veiga function,[4] which models the absorption factor A as an exponential of a polynomial in terms of wavelength, with coefficients defined by polynomials of $\sin(\theta)$. These methods are good with balancing accuracy with efficiency but still require the evaluation of relatively complex expressions.

In contrast, our methods avoid both standalone approximation and integration completely. We introduce a fast approximate calculator of A^* for a given μR, which relies on a single pre-computed reference A curve and polynomial interpolation for efficient evaluation. This approach reduces the computation time to around 10^{-4} seconds for a single curve. For benchmarking, we compare against the *International Tables*,[5] which uses a double integration based on cosh, our simplified summation-based method achieved comparable results for $2\theta \leq 140^\circ$ with deviations of $\leq 0.12\%$ and $\leq 0.35\%$ for for $0.25 \leq \mu R \leq 3.5$ and $3.5 \leq \mu R \leq 10$ for both our brute-force approach and/or polynomial fits.

We now describe how we come up with our fast calculation as well as a comparison to our

brute-force approach for a general evaluation. We confine the estimates to $0.25 \leq \mu R \leq 3.5$ as this range is the most commonly used. As a start, we explore estimating A^* through an interpolative reconstruction approach using polynomials, given a known value for μR. Our strategy is to build a relatively sparse database of A^* curves computed by brute force, and to find curves for μR values in between using interpolation of polynomials. The $1/A^*$ curves vary less strongly than A^*, with all values falling between 0 and 1, and we found the interpolation works better on $1/A^*$ than on A^* itself.

We started by creating a sparse dataset of $1/A^*$ curves for μR = 0.25, 0.5, 1, 1.5, 2, 2.5, 3, and 3.5 over a range $1 \leq 2\theta \leq 180°$ on a grid of spacing of $\Delta 2\theta = 0.1°$ using the brute-force approach. We then carried out a fit of a polynomial to each of these curves. After experimenting with using different polynomial degrees, we found that a sixth-degree polynomial provided reliable reconstructions. The reconstructed curves are very close to the original ones as can be seen in Fig. S2(a) which shows a comparison on the brute force and polynomial fits for the A^* curves chosen above. The fits are very good, as the blue brute-force curves are barely visible. Small errors are seen clustered around low 2θ, with maximum percentage error of around 0.68% for $\mu R = 2.5$ at $2\theta = 1°$. The maximum percentage errors for μR = 2, 2.5, and 3 also occurred at $2\theta = 1°$, with values of 0.03%, 0.12%, 0.32%, respectively. For $\mu R \leq 1.5$, the errors are almost negligible, with the maximal percentage error much less than $\leq 10^{-2}$%. The polynomial coefficients appropriate to each μR are then stored in a database.

We then need a method to find the A^* curves for any μR that may lie between those in our sparse database. To interpolate between curves of different μR, we used quadratic interpolation for the polynomial coefficients. We also experimented with linear and cubic interpolations: the former is not accurate enough and the latter tends to over-fit, settling on quadratic interpolation as the best approach. The full A^* curve is then reconstructed using the resulting polynomial function. The accuracy of this approach is illustrated in Fig. S2 (b). In the figure the blue curves are computed for intermediate μR values using the full

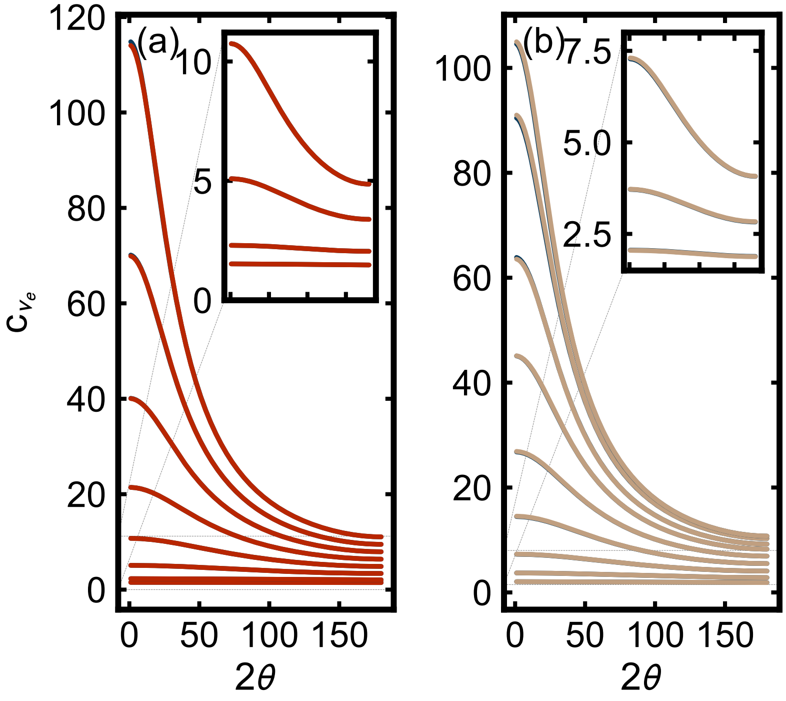

Figure S2: Comparison of brute-force and fast calculation of A^*. (a) Polynomial fits (red) and brute-force-calculated curves (blue, almost invisible) for μR values between 0.25 and 3.5 from our database of curves. (b) Polynomial curves found by interpolation from the database curves (brown), and brute-force curves computed for the same μR values (blue, almost invisible), for various μR's randomly chosen between μR's in the sparse dataset. In both panels, the insets show curves for smaller μR's on an expanded scale. From bottom to top in (a) $\mu R = 0.25$, 0.5, 1, 1.5, 2, 2.5, 3, and 3.5, and in (b) $\mu R = 0.4319$, 0.7987, 1.235, 1.71, 2.172, 2.6, 2.912, 3.25, and 3.4.

brute force calculation, and the light brown curve is the one estimated using the polynomial interpolation approach. Since the maximum percentage errors are $< 1.1\%$ for all μR's, we conclude that our model provides highly accurate estimations for A^* across various μR values within our chosen range of $0.25 \leq \mu R \leq 3.5$.

To summarize, we take x as the $1/A^*$ curve for $\mu R = 0.5$. The functional form is $A^*(x) = 1/\sum_{i=0}^{6} a_i x^i$. For the selected set of μR's, the corresponding coefficients a_i's are listed in Table S3. For all other μR's in between, e.g. μR_0, we interpolate each coefficient a_i using the quadratic form $b_i = \alpha_i(\mu R_0)^2 + \beta_i(\mu R_0) + \gamma_i$, where the constants α_i, β_i, and γ_i are determined from $b_i = \alpha_i(\mu R_j)^2 + \beta_i(\mu R_j) + \gamma_i$ for $j = 1, 2, 3$, corresponding to the three closest μR's to μR_0. The interpolated function is then $A^*_{\mu R_0}(x) = 1/\sum_{i=0}^{6} b_i x^i$.

Table S3: Polynomial fit coefficients a_i for selected values of μR.

μR	0.25	0.5	1	1.5	2	2.5	3	3.5
a_0	−178.71	0.00	−234.76	−4803.17	−12928.52	−21220.24	−27207.09	−30121.29
a_1	2378.16	1.00	1912.08	58944.34	162014.94	267802.21	344395.55	381710.23
a_2	−13126.53	0.00	−4395.40	−300757.71	−845218.60	−1407243.86	−1815244.44	−2014105.86
a_3	38603.19	0.00	−4028.75	816333.28	2349291.87	3940828.56	5099123.96	5663734.05
a_4	−63802.11	0.00	33089.88	−1242527.99	−3668761.30	−6202335.41	−8050708.34	−8951452.33
a_5	56203.20	0.00	−51225.01	1005023.63	3051609.21	5201294.21	6773264.75	7538833.21
a_6	−20619.13	0.00	26505.59	−337279.32	−1056051.28	−1815539.12	−2372171.98	−2642993.45

S4 Estimation of μR using model fitting of z-scans

Here we develop a model for estimating μR experimentally from z-scan data. We define the x direction as the direction of travel of the x-ray beam and z to be the vertical direction perpendicular to the capillary (see Fig. 4 in the main paper). We place the origin at the center of the x-ray beam. Let $R = D/2$ be the radius of the capillary cross-section, and h to be the height of the x-ray beam. Finally, we define z_0 to be the height of the center of the capillary. z_0 could take any value without loss of generality but for convenience we try and set $z_0 \sim 0$. This offset will be refined in the fit.

The path length through the sample of an x-ray ray at the height, z is then given by

$$l = \begin{cases} 2\sqrt{R^2 - (z - z_0)^2} & \text{for } |z - z_0| \leq R \\ 0 & \text{otherwise.} \end{cases} \tag{29}$$

We define I_0 as the intensity of the unattenuated x-ray beam (assumed to have a uniform cross-section). We found that, in general, I_0 had a small, approximately linear, dependence on z, so we also define a coefficient, m, to account for this to obtain the best fits. In addition, we assume z is sampled at discrete intervals with a constant step size Δz.

We therefore define the intensity function $I[z]$ as

$$I[z] = \begin{cases} I_0 \cdot e^{-2\mu\sqrt{R^2-(z-z_0)^2}} + mz & \text{for } |z - z_0| \leq R \\ I_0 + mz & \text{otherwise.} \end{cases} \tag{30}$$

The normalized kernel function $K[z]$ to account for the finite width beam is given by

$$K[z] = \frac{1}{C} \begin{cases} 1 & \text{for } |z| \leq h/2 \\ 0 & \text{otherwise,} \end{cases} \tag{31}$$

where C is a constant such that $\sum_z K[z] = 1$. Therefore, C is the number of z such that $|z| \leq h/2$, and $C = 2\lfloor \frac{h}{2\Delta z} \rfloor + 1$.

To fit the measured curves we need to convolute $I[z]$ with $K[z]$, denoted as $(I \circledast K)[z]$,

given by

$$
\begin{aligned}
(I \circledast K)[z] &= \sum_t I[t] \cdot K[z-t] && (32)\\
&= \sum_t I[t] \begin{cases} \frac{1}{C} & \text{for } |z-t| \leq h/2 \\ 0 & \text{otherwise} \end{cases} && (33)\\
&= \frac{1}{C} \sum_{t:|z-t|\leq h/2} I[t] && (34)\\
&= \frac{1}{C} \sum_{t:|z-t|\leq h/2} \begin{cases} I_0 \cdot e^{-2\mu\sqrt{R^2-(t-z_0)^2}} + mt & \text{for } |t-z_0| \leq R \\ I_0 + mt & \text{otherwise} \end{cases} && (35)\\
&= \frac{1}{C} \sum_{k=1}^{C} \begin{cases} I_0 \cdot e^{-2\mu\sqrt{R^2-(t_k-z_0)^2}} + mt_k & \text{for } |t_k-z_0| \leq R \\ I_0 + mt_k & \text{otherwise,} \end{cases} && (36)
\end{aligned}
$$

where $t_k \in [z-h/2, z+h/2]$ with $t_{i+1} = t_i + \Delta z$ for $i = 1, 2, ..., C-1$.

As seen from Eq. 36, the convolution at each z is essentially an average of $I[t_k]$ for each $t_k \in [z-h/2, z+h/2]$. As h increases, the convolution deviates more significantly from the intensity function in Eq. 30 and as $h \to 0$, $C \to 1$ meaning that the number of t_k we are averaging approaches 1, in which case, Eq. 36 is just the unconvoluted intensity, Eq. 30.

We have developed a user-friendly fitting program in DIFFPY.UTILS[6] that accurately and efficiently extracts μR by optimizing the six parameters: μ, D, h, I_0, z_0, and m. The function only takes in a set of experimental z and I values and minimizes the sum of squared residuals between the experimental data and the model convolution. The convolution is computed using SciPy signal's convolve function with same mode, based on the intensity and kernel functions defined in Eq. 30 and Eq. 31. Boundary effects occur because the kernel only partially overlaps with the intensity signal at the edges, resulting in incomplete averaging. To address this, we extend the z values for both intensity and kernel functions to ensure full coverage for the given z values. The same mode also introduces a potential

shift in convolution as it crops the result to match the size of z. As a result, we recenter the kernel around $z - \bar{z}$, where $\bar{z}$ is the average of z, aligning the intensity with the convolution. To optimize the search for the global minimum, we use SciPy's dual annealing method, a stochastic global optimization algorithm. This approach provides a robust solution to find the global minimum. By combining the theoretical results from Eq. 30, Eq. 31, and Eq. 36 with the fitting program, we achieve a more accurate estimation of μR.

S5 Estimation of μR using a theoretical database

The process for estimating the theoretical linear attenuation coefficient μ involves using the XrayDB database,[7] which can then be used to compute μR by multiplying with the capillary radius R. The inputs required for this calculation are the sample composition, the x-ray energy, and either a measured mass density, ρ_s, for the sample, or the packing fraction f, where $\rho_s = \rho_m \cdot f$, where ρ_m is the mass density of fully dense sample that can be estimated from the atomic structure if it is known.

We briefly reproduce the computational process here, for which a similar description can be found in Lipp *et al.*[8] To calculate μ, we first compute the mass attenuation coefficient $\left(\frac{\mu}{\rho}\right)_e$ for each element e in the sample, in cm^2/g. A mass-weighted sum approach is then applied to determine the total mass attenuation coefficient for the mixture. Let E be the set of sample elements in the sample, f_e be the number of atoms per element e, and A_e be its atomic mass in g/mol. Then the mass contribution of each element e is given by $m_e = f_e \cdot A_e$, from which the total mass attenuation coefficient $\left(\frac{\mu}{\rho}\right)_{\mathrm{tol}}$ and total mass m_{tol} are computed as

$$\left(\frac{\mu}{\rho}\right)_{\mathrm{tol}} = \sum_{e \in E} \left(\frac{\mu}{\rho}\right)_e \cdot m_e \tag{37}$$

$$m_{\mathrm{tol}} = \sum_{e \in E} m_e. \tag{38}$$

The final μ value is then obtained by normalizing $\left(\frac{\mu}{\rho}\right)_{\mathrm{tol}}$ by m_{tol} and scaling by the sample mass density ρ_s:

$$\mu = \rho_s \cdot \frac{\left(\frac{\mu}{\rho}\right)_{\mathrm{tol}}}{m_{\mathrm{tol}}}. \tag{39}$$

S6 Selecting μR_e in different situations

In the main paper we assessed the effect of different A^* corrections on refined structural parameters from models. Here, for completeness, we present more complete results of this study by taking the optimal μR corrections but applying them to data from different thickness CeO_2 samples with different degrees of absorption.

In Table S4 we show the refined parameters for synchrotron, uncorrected and best-corrected data for all CeO_2 datasets, with additional μR's tested listed in Tables S5, S6, and S7. We find that for data collected with IDs 0.813 mm and 1.024 mm here, the μR_e's, 3 and 5.5, are larger than their theoretical values, 2.28 and 2.91, respectively. The ratio $\mu R_e/\mu R_{th}$ increases from about 1.3 to almost 2 as μR_{th} increases. In both cases, the optimally corrected data resulted in an R_w decreasing by half from the uncorrected data. For data with ID = 0.635 mm, $\mu R_e = 1.53$, which is smaller than $\mu R_{th} = 2.11$. Notably, this value for μR_e was obtained from the z-scan absorption measurement that was made with the slit and detector in the experimental condition, except with reduced channels.

For moderate $\mu R_{th} \leq 2.2$, if it is possible to measure sample absorption using a z-scan and using the fitting program in our DIFFPY.LABPDFPROC software to extract μR, it is recommended to do this with the slits for the z-scan in the same settings as was used to collect the experimental data, but with reduced channels. However, if it is not possible, μR_e can be approximated by scaling the theoretical value μR_{th} by about 80% to get an approximately correct value for the effective μR to use in the A^* calculation. For higher $\mu R > 2.2$ we recommend testing μR's between 1.3-2$\times\mu R_{th}$. Future investigations with more

data will be necessary to confirm these ranges and better understand the behavior.

Table S4: Results of fittings for CeO_2 synchrotron, uncorrected, and best-corrected data, over $r_{\mathrm{min}} = 1.0$ and $r_{\mathrm{max}} = 40.0$. The uncorrected data are listed with their IDs (1.024 mm, 0.813 mm, and 0.635 mm), and the best-corrected data are labeled with the ID followed by "corrected".

Parameter	synchrotron	0.635 mm	0.813 mm	1.024 mm	0.635 mm corrected	0.813 mm corrected	1.024 mm corrected
s_1	0.37434(21)	0.3919(13)	0.2992(12)	0.2206(10)	0.4684(13)	0.4438(14)	0.3500(11)
Q_{damp}	0.02386(4)	0.02831(20)	0.03019(25)	0.03211(27)	0.02672(19)	0.02616(22)	0.02262(25)
Q_{broad}	0.01814(7)	0.0246(4)	0.0267(5)	0.0256(5)	0.02820(35)	0.0310(4)	0.0389(4)
δ_2	9.07(4)	12.962(35)	8.79(22)	8.82(31)	7.92(11)	7.13(9)	7.282(11)
a	5.414232(7)	5.40344(5)	5.40214(5)	5.40198(6)	5.40277(4)	5.40037(5)	5.39897(6)
Ce(U_{iso})	0.003571(5)	0.003028(24)	0.002381(24)	0.002117(24)	0.003365(24)	0.003471(28)	0.003453(29)
O(U_{iso})	0.04120(10)	0.0854(13)	0.0999(23)	0.123(4)	0.0643(8)	0.0523(7)	0.0411(5)
Q_{max}	30.0	16.6	16.6	16.6	16.6	16.6	16.6
grid	0.10472	0.189253	0.189253	0.189253	0.189253	0.189253	0.189253
R_w	0.160823	0.492458	0.627628	0.735509	0.355323	0.320432	0.367162
χ^2_{red}	961.528958	667.00816	779.978787	1097.63411	390.090295	254.331573	337.490905

Table S5: Results of the refinement for CeO_2 data (ID=0.635 mm) for synchrotron, uncorrected, and corrected data with different μR's, over $r_{\mathrm{min}} = 1.0$ and $r_{\mathrm{max}} = 40.0$. The μR's are the values used for each correction.

Parameter	synchrotron	uncorrected	$\mu R = 0.97$	$\mu R = 1.53$	$\mu R = 1.87$	$\mu R = 2$	$\mu R = 2.11$	$\mu R = 5$
s_1	0.37434(21)	0.3919(13)	0.4379(13)	0.4698(13)	0.4793(13)	0.4379(5)	0.4817(12)	0.3607(6)
Q_{damp}	0.02386(4)	0.02831(20)	0.02787(20)	0.02700(19)	0.02623(18)	0.02090(9)	0.02571(18)	0.02025(15)
Q_{broad}	0.01814(7)	0.0246(4)	0.0243(4)	0.02415(34)	0.02410(33)	0.01343(11)	0.02821(32)	0.02728(20)
δ_2	9.07(4)	12.962(35)	13.322(21)	13.589(14)	13.733(10)	1.020(31)	7.34(10)	4.19(6)
a	5.414232(7)	5.40344(5)	5.40313(4)	5.40286(4)	5.40271(4)	5.40568(4)	5.40248(4)	5.40203(4)
Ce(U_{iso})	0.003571(5)	0.003028(24)	0.003323(24)	0.003725(26)	0.004047(27)	0.07334(12)	0.003897(26)	0.00879(4)
O(U_{iso})	0.04120(10)	0.0854(13)	0.0783(11)	0.0717(9)	0.0683(8)	0.2377(7)	0.0572(6)	0.0579(4)
Q_{max}	30.0	16.6	16.6	16.6	16.6	16.6	16.6	16.6
grid	0.10472	0.189253	0.189253	0.189253	0.189253	0.189253	0.189253	0.189253
R_w	0.160823	0.492458	0.428941	0.355725	0.309613	0.285437	0.276858	0.227684
χ^2_{red}	961.528958	667.00816	525.159995	390.974207	318.669272	1895.392085	271.44589	387.786201

Table S6: Results of the refinement for CeO_2 data (ID=0.813 mm) for synchrotron, uncorrected, and corrected data with different μR's, over $r_{\text{min}} = 1.0$ and $r_{\text{max}} = 40.0$. The μR's are the values used for each correction.

Parameter	synchrotron	uncorrected	$\mu R = 2.5$	$\mu R = 3$	$\mu R = 3.5$	$\mu R = 4$	$\mu R = 4.5$	$\mu R = 5$
s_1	0.37434(21)	0.2992(12)	0.4411(14)	0.4438(14)	0.4301(12)	0.4101(11)	0.3893(10)	0.3705(9)
Q_{damp}	0.02386(4)	0.03019(25)	0.02720(22)	0.02615(22)	0.02474(21)	0.02334(21)	0.02202(21)	0.02076(22)
Q_{broad}	0.01814(7)	0.0267(5)	0.0297(4)	0.0311(4)	0.0320(4)	0.03281(35)	0.03356(34)	0.03468(34)
δ_2	9.07(4)	8.79(22)	7.65(13)	7.13(9)	7.10(8)	7.04(7)	6.85(7)	6.26(10)
a	5.414232(7)	5.40214(5)	5.40072(5)	5.40037(5)	5.40010(5)	5.39988(5)	5.39971(5)	5.39955(5)
Ce(U_{iso})	0.003571(5)	0.002381(24)	0.003181(28)	0.003470(28)	0.003821(30)	0.004188(31)	0.004571(33)	0.00491(4)
O(U_{iso})	0.04120(10)	0.0999(23)	0.0580(8)	0.0522(7)	0.0491(6)	0.0474(5)	0.0465(5)	0.0457(5)
Q_{max}	30.0	16.6	16.6	16.6	16.6	16.6	16.6	16.6
grid	0.10472	0.189253	0.189253	0.189253	0.189253	0.189253	0.189253	0.189253
R_w	0.160823	0.627628	0.373648	0.320432	0.286607	0.271127	0.269108	0.274893
χ^2_{red}	961.528958	779.978787	316.35313	254.331591	224.314884	220.418871	237.302097	268.437308

Table S7: Results of the refinement for CeO_2 data (ID=1.024 mm) for synchrotron, uncorrected, and corrected data with different μR's, over $r_{\text{min}} = 1.0$ and $r_{\text{max}} = 40.0$. The μR's are the values used for each correction.

Parameter	synchrotron	uncorrected	$\mu R = 3.5$	$\mu R = 4$	$\mu R = 4.5$	$\mu R = 5$	$\mu R = 5.5$	$\mu R = 7.5$
s_1	0.37434(21)	0.2206(10)	0.3857(14)	0.3849(13)	0.3757(12)	0.3636(11)	0.3514(11)	0.3079(9)
Q_{damp}	0.02386(4)	0.03211(27)	0.02790(24)	0.02683(24)	0.02545(24)	0.02407(25)	0.02268(25)	0.01743(29)
Q_{broad}	0.01814(7)	0.0257(5)	0.0318(5)	0.0339(5)	0.0357(4)	0.0376(4)	0.0402(4)	0.0471(5)
δ_2	9.07(4)	8.82(31)	7.49(15)	7.12(11)	7.08(10)	6.97(9)	6.44(12)	4.91(15)
a	5.414232(7)	5.40197(6)	5.39991(6)	5.39959(6)	5.39933(6)	5.39910(6)	5.39889(6)	5.39831(6)
Ce(U_{iso})	0.003571(5)	0.002116(24)	0.002948(29)	0.003076(29)	0.003209(30)	0.003327(30)	0.003361(32)	0.00367(4)
O(U_{iso})	0.04120(10)	0.122(4)	0.0516(8)	0.0474(7)	0.0446(6)	0.0423(6)	0.0398(5)	0.0351(5)
Q_{max}	30.0	16.6	16.6	16.6	16.6	16.6	16.6	16.6
grid	0.10472	0.189253	0.189253	0.189253	0.189253	0.189253	0.189253	0.189253
R_w	0.160823	0.735509	0.431096	0.40035	0.381211	0.370642	0.367111	0.376751
χ^2_{red}	961.528958	1097.634093	371.18157	338.362728	324.657538	324.015065	337.397013	418.954406

S7 Refinement across extended μR values

For a low $\mu R < 1$, the uncorrected lab data already provides a reasonably good fit (low R_w). This is demonstrated in Table S8 for ZrO_2 packed in a 1.024 mm capillary, where the theoretical μR is ~ 0.7. We have applied two corrections, corresponding to the largest and smallest experimental μR's computed from Method 2, using $h = 0.05$ mm and 1.2 mm, respectively, with a reduced channel. To avoid overfitting, isotropic ADPs were used for zirconium and oxygen.

Overall, we find that the uncorrected lab data provides results comparable to the synchrotron data. As with the ceria data described in the main paper, the lattice parameters of the synchrotron data are slightly overestimated. In ceria, the ADP on the metal site was most important. Here the difference in scattering power between oxygen and Zr is smaller and the oxygen ADPs are more significant. We find that the ADPs of the metal increase, while those of of the oxygen decrease, and R_w decreases with increasing absorption correction. The refinements are quite reproducible between the synchrotron and lab data, considering the significant differences between the resolutions and Q-ranges of the different measurements, but do not allow a clear differentiation between the different absorption corrections.

The PDFs of the synchrotron and laboratory data are plotted in Fig. S9. The most significant difference is that the lab data have slightly broader peaks compared to the synchrotron data due to the lower Q_{max}, but all the fits are of high quality.

Table S8: Results of fittings for ZrO_2 data (ID=1.024 mm) for synchrotron, uncorrected, and corrected lab data, over $r_{\mathrm{min}} = 1.0$ and $r_{\mathrm{max}} = 40.0$. The μR's are the values applied for correction.

Parameter	synchrotron	uncorrected	$\mu R = 0.56$	$\mu R = 0.76$
s_1	0.49519(28)	0.3849(26)	0.4022(26)	0.4225(27)
Q_{damp}	0.02282(5)	0.0251(5)	0.0239(5)	0.0234(5)
Q_{broad}	0.03182(13)	0.0366(13)	0.0365(13)	0.0364(12)
δ_2	1.294(17)	2.90(10)	2.87(9)	2.83(9)
a	5.148597(23)	5.1455(4)	5.1457(4)	5.1457(4)
b	5.211724(23)	5.2065(4)	5.2068(4)	5.2070(4)
c	5.317103(24)	5.3091(4)	5.3094(4)	5.3096(4)
β	99.2208(4)	99.201(7)	99.202(7)	99.203(7)
Zr(U_{iso})	0.0010703(25)	0.00281(7)	0.00291(7)	0.00300(7)
O(U_{iso})	0.03721(12)	0.0360(13)	0.0355(12)	0.0355(12)
Q_{max}	30.0	16.6	16.6	16.6
grid	0.10472	0.189253	0.189253	0.189253
R_w	0.218742	0.30822	0.301004	0.290563
χ^2_{red}	1298.813481	36.992841	38.263451	38.131731

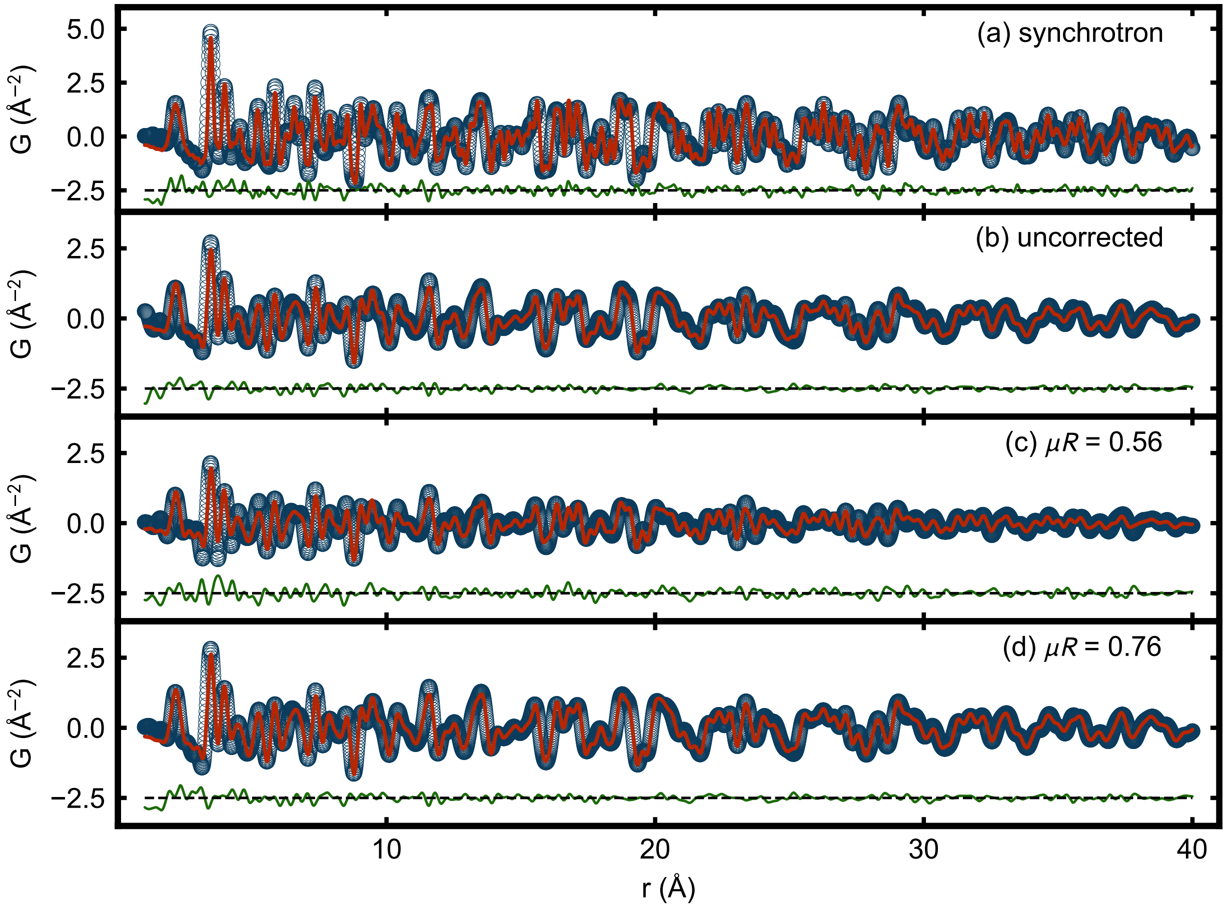

Figure S9: Results of ZrO_2 synchrotron, uncorrected, and corrected laboratory data over $r_{\mathrm{min}} = 1.0$ and $r_{\mathrm{max}} = 40.0$. The μR's reported inside the panels are the values used for correction. The measured PDFs are the blue circles, the best-fit PDFs are the red lines, and the residuals shown are green.

We now turn to the case of very high μR by considering data from the HfO_2 sample. As discussed in the main paper, the significant absorption of the sample results in a strong suppression of the diffraction signal, resulting in a very low signal/noise ratio. This is evident by looking at the dark blue curve in Fig. S10(a), which shows the raw XRD data for a measurement of HfO_2 using a capillary of 0.635 mm, where the theoretical μR is just above 4. As shown in the inset, only a few weak signals are observed, and the curve remains relatively flat until the end, where there is a strong upturn. While the absorption correction would adjust signal amplitudes, it amplifies both the signals and the noise by the same amount, leaving the signal/noise ratio unaffected. As a result, the data is functionally

useless for PDF analysis.

It is possible to load the sample into a thinner capillary if one is available. However, a simple workaround is to take a glass or plastic wire and rub some grease and powder on the outside of the wire (see Figure S11). This approach is very effective if the powder scatters well enough, which is typically the case for elements with large x-ray cross-sections. In Fig. S10 (a), the raw XRD data for the wire is shown in light blue. Comparing it to the dark blue curve (representing HfO_2 in the 0.635 mm capillary), the wire data shows a significant improvement in the signal/noise ratio, with a reduced upturn at the end, indicating much better data quality. To evaluate the effectiveness further, Fig. S10 (b) shows the PDF obtained from the polarization corrected wire diffraction pattern, fitted between $r_{\mathrm{min}} = 1.0$ and $r_{\mathrm{max}} = 20.0$ Å. The refined parameters are provided in Table S12. The small residuals of the fit confirm the high data quality, making the wire data the preferred choice.

Finally, we present a method to estimate the theoretical μR for wire data, which is useful for determining the effective μR to use to apply for the absorption correction. Direct theoretical estimation is impossible since we are using a wire instead of a capillary, meaning that we do not know the powder density and exact diameter of the wire and powder together. As a result, we can use a z-scan to obtain experimental values, using, for instance, a small x-ray beam height of 0.1 mm with a reduced channel. This returns an experimental μR value close to the theoretical one. Fig. S10 (c) shows the z-scan fit using Method 2 with the experimental settings mentioned above. Although the fitted red curve shows some deviation from the original blue intensity data, making it less accurate, we find it still reliable, as verified through both Method 1 ($\mu R_{m1} = 0.39$) and Method 2 ($\mu R_{m2} = 0.35$).

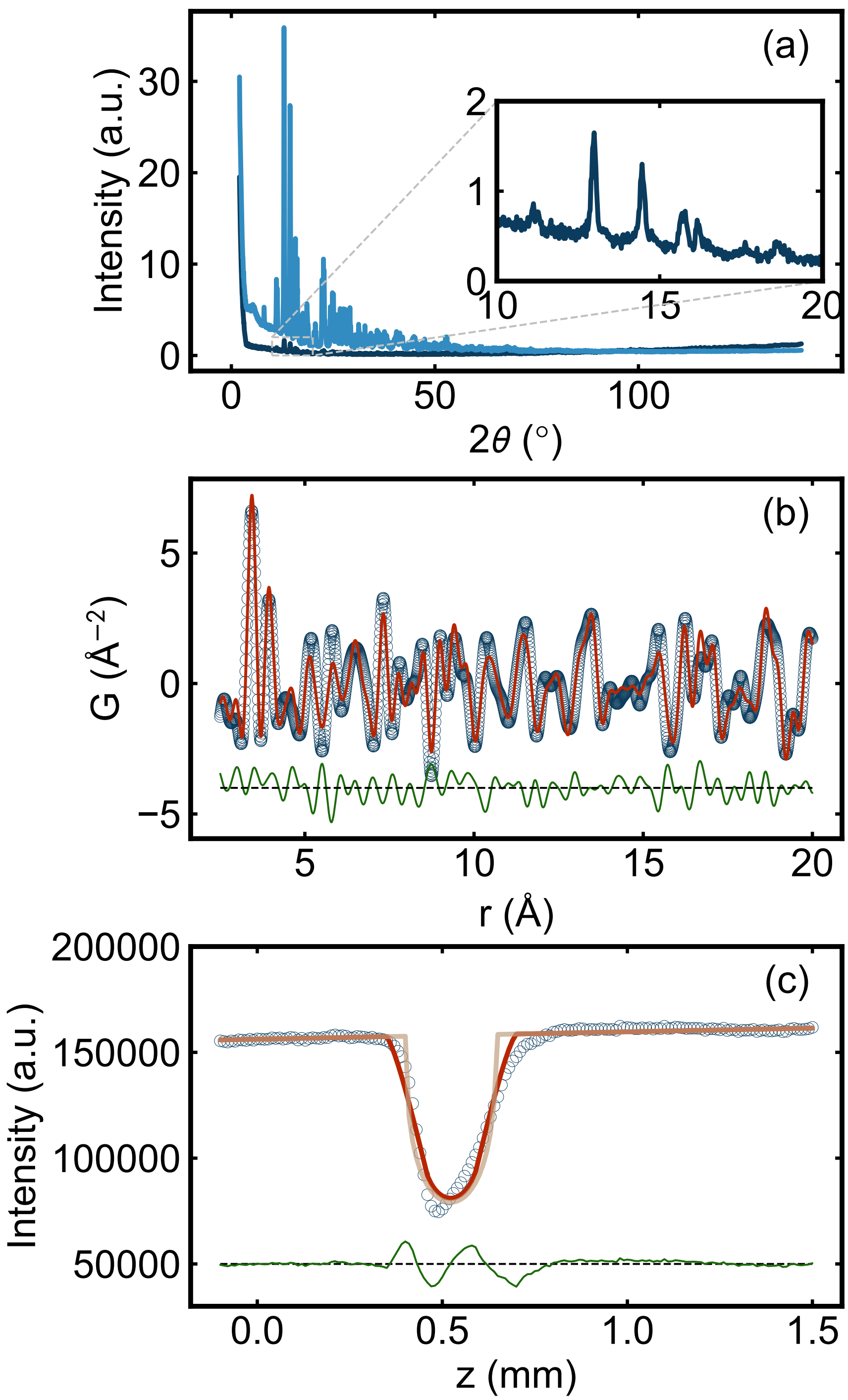

Figure S10: XRD, PDF, and z-scan analysis of HfO_2 data. (a) raw XRD comparison of HfO_2 measured with a capillary (dark blue) and wire (light blue), with an inset showing an expanded portion of the capillary data highlighting its only signals. (b) PDF fit for wire data with only polarization correction applied, showing the measured PDF (blue), the best-fit PDF (red), and residuals (green), fitted between $r_{\mathrm{min}} = 1.0$ and $r_{\mathrm{max}} = 20.0$. (c) z-scan fit for wire data, with the original intensity (blue), the fit (red), the unconvoluted intensity (brown), and residuals (green).

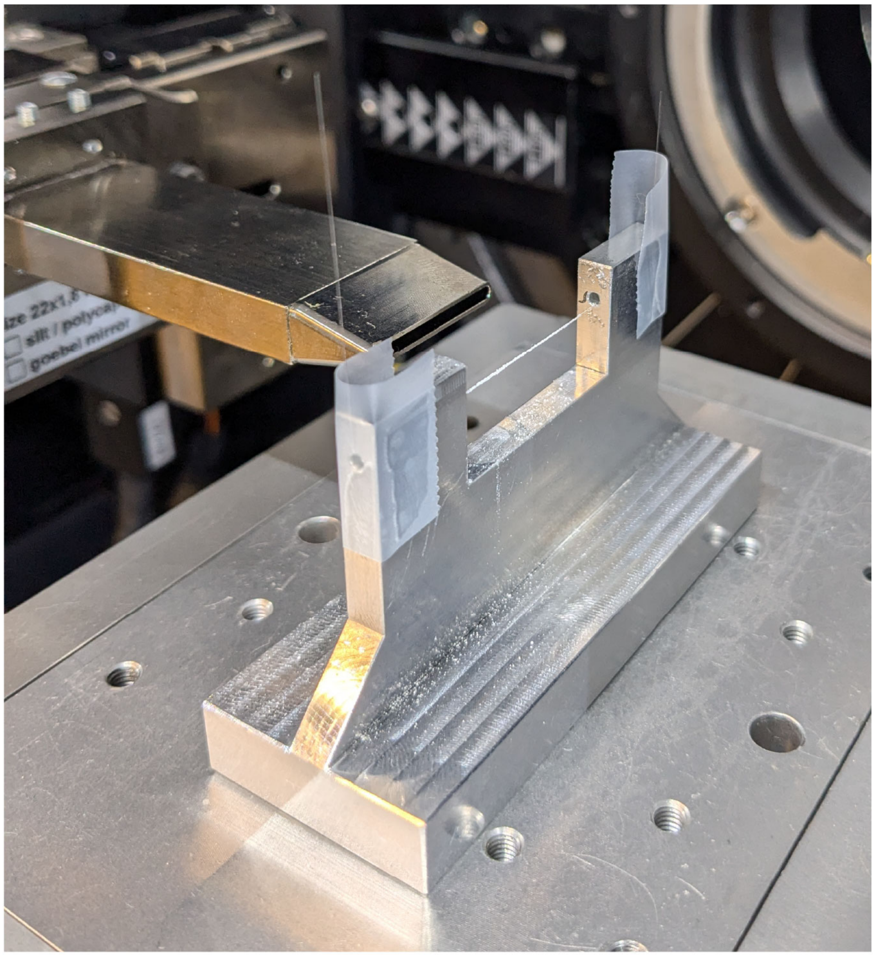

Figure S11: Photograph of the wire coated with the powder of HfO_2. The wire was coated with some grease, then, the hafnia powder was dispersed over the coated area.

Table S12: Results of the refinement for uncorrected HfO_2 wire data over $r_{\text{min}} = 1.0$ and $r_{\text{max}} = 20.0$.

Parameter	Uncorrected
s_1	0.714(15)
Q_{damp}	0.025(5)
Q_{broad}	0.047(8)
δ_2	6.84(11)
a	5.1128(16)
b	5.1716(16)
c	5.2836(19)
β	99.26(4)
U11_0	0.0051(5)
U22_0	0.00209(24)
U33_0	0.0057(6)
U12_0	0.00238(27)
U13_0	0.00211(26)
U23_0	0.0041(4)
U11_4	0.052(12)
U22_4	0.056(13)
U33_4	0.070(17)
U12_4	0.056(13)
U13_4	0.064(14)
U23_4	0.062(15)
U11_8	0.033(6)
U22_8	0.021(4)
U33_8	0.102(15)
U12_8	0.035(5)
U13_8	0.064(10)
U23_8	0.058(8)
x_0	0.27352(28)
y_0	0.45880(31)
z_0	0.7072(5)
x_4	0.160(5)
y_4	0.242(5)
z_4	0.873(5)
x_8	0.4723(20)
y_8	0.7220(15)
z_8	0.977(4)
Q_{max}	16.6
grid	0.189253
R_w	0.22502
χ^2_{red}	12.402705

S8 Additional Tables and Figures

Table S13: List of μR's computed using the four different methods described in the texts, labeled as Method 1, Method 2, Method 3, and Method 4. "Reduced" and "Open" channel conditions correspond to 3 and 191 open active channels, respectively.

Sample	ID (mm)	Density (g/cm^3)	h (mm)	Channel Condition	Method 1	Method 2	Method 3	Method 4
ZrO_2	0.635	1.009	0.10	Reduced	0.31	0.27	0.26	0.40
	0.813	0.856	0.10	Reduced	0.36	0.30	0.30	0.43
	1.024	1.122	0.05	Reduced	0.78	0.76	0.77	0.71
			0.20	Reduced	0.70	0.70	0.71	
			0.60	Reduced	0.56	0.56	0.60	
			1.20	Reduced	0.57	0.56	0.60	
CeO_2	0.635	1.706	0.05	Reduced	1.87	1.88	1.86	2.11
			0.10	Reduced	1.86	1.81	1.81	
			0.20	Reduced	1.76	1.70	0.71	
			0.60	Reduced	1.55	1.49	1.64	
			1.00	Reduced	1.53	1.47	1.63	
			0.05	Open	1.64	1.60	1.58	
			0.20	Open	1.50	1.45	1.44	
			0.60	Open	0.99	0.94	0.98	
			1.00	Open	0.96	0.94	0.97	
	0.813	1.435	0.10	Reduced	2.27	2.24	2.26	2.28
	1.024	1.457	0.10	Reduced	2.71	2.66	2.70	2.91
HfO_2	0.635	1.741	–	–	–	–	–	4.08
	0.813	1.963	0.10	Open	2.95	2.78	2.77	5.90
	Wire	–	0.10	Reduced	0.39	0.35	–	–

Table S14: List of fitted and theoretical or given physical parameters μ, D, and h. $\mu_{\text{theoretical}}$ is the theoretical μ computed from XrayDB, D_{given} is the given inner diameter (ID) of each capillary, h_{given} is the given x-ray beam height. μ_{fit}, D_{fit}, and h_{fit} are the fitted parameters computed from Method 2 for each z-scan data. "Reduced" and "Open" channel conditions correspond to 3 and 191 open active channels, respectively.

Sample	D_{given} (mm)	Density (g/cm^3)	h_{given} (mm)	Channel	$\mu_{\text{theoretical}}$ (mm^{-1})	μ_{fit} (mm^{-1})	D_{fit} (mm)	h_{fit} (mm)
ZrO_2	0.635	1.009	0.10	Reduced	1.252	0.816	0.660	0.094
	0.813	0.856	0.10	Reduced	1.062	0.743	0.813	0.057
	1.024	1.122	0.05	Reduced	1.392	1.503	1.016	0.076
			0.20	Reduced		1.387	1.008	0.174
			0.60	Reduced		1.177	0.951	0.297
			1.20	Reduced		1.172	0.962	0.283
CeO_2	0.635	1.706	0.05	Reduced	6.659	5.857	0.641	0.073
			0.10	Reduced		5.690	0.635	0.077
			0.20	Reduced		5.388	0.631	0.150
			0.60	Reduced		5.155	0.577	0.277
			1.00	Reduced		5.133	0.574	0.264
			0.05	Open		4.989	0.642	0.092
			0.20	Open		4.530	0.640	0.166
			0.60	Open		3.100	0.608	0.337
			1.00	Open		3.065	0.611	0.357
	0.813	1.435	0.10	Reduced	5.601	5.555	0.805	0.067
	1.024	1.457	0.10	Reduced	5.687	5.265	1.012	0.074
HfO_2	0.635	1.741	–	–	12.86	–	–	–
	0.813	1.963	0.10	Open	14.50	6.823	0.814	0.084
	Wire	–	0.10	Reduced	–	2.855	0.246	0.103

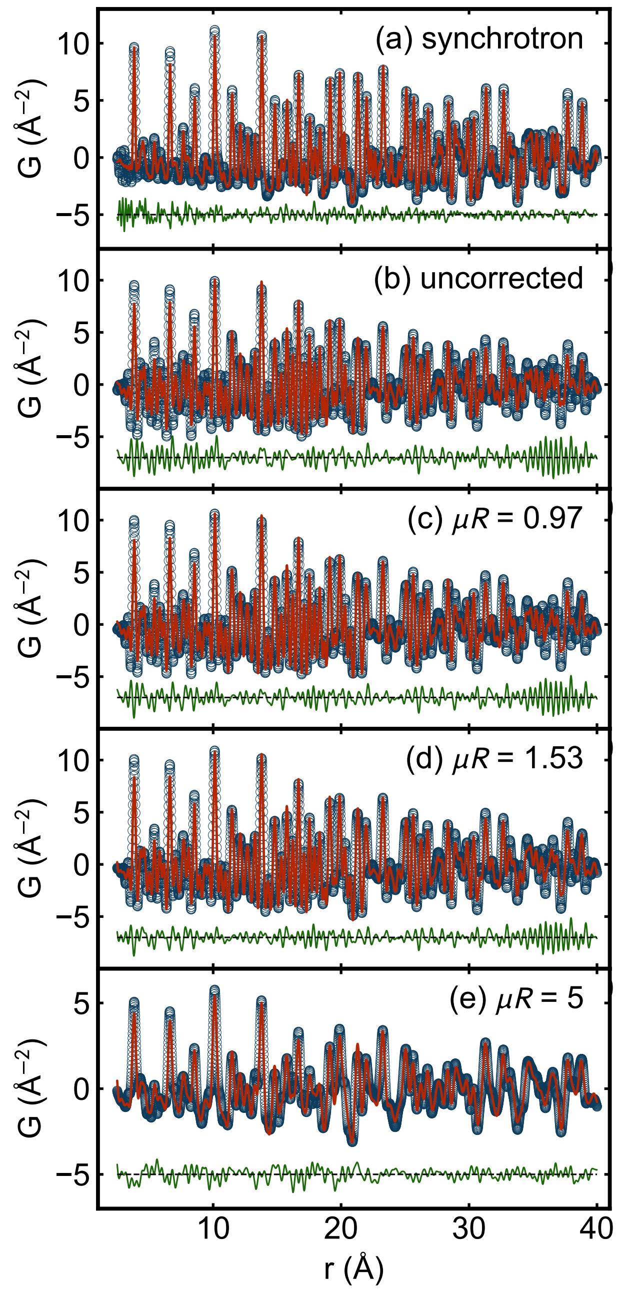

Figure S15: CeO_2 PDF fits for synchrotron, uncorrected, and corrected data with different μR's (reported in the panels), over $r_{\mathrm{min}} = 1.0$ and $r_{\mathrm{max}} = 40.0$. The plots show the measured PDFs (blue), the best-fit PDFs (red), and the residuals (green).

References

(1) Billinge, S. J. L.; Farrow, C. L. Towards a Robust Ad Hoc Data Correction Approach That Yields Reliable Atomic Pair Distribution Functions from Powder Diffraction Data. *Journal of Physics: Condensed Matter* **2013**, *25*, 454202.

(2) Dwiggins Jnr, C. W. Rapid Calculation of X-ray Absorption Correction Factors for Cylinders to an Accuracy of 0.1%. *Acta Crystallographica Section A: Crystal Physics, Diffraction, Theoretical and General Crystallography* **1975**, *31*, 146–148.

(3) Ida, T. Efficiency in the Calculation of Absorption Corrections for Cylinders. *Journal of Applied Crystallography* **2010**, *43*, 1124–1125.

(4) Larson, A. C.; Dreele, R. B. V. GENERAL STRUCTURE ANALYSIS SYSTEM.

(5) Maslen, E. N. In *International Tables for Crystallography*; Wilson, A. J. C., Ed.; Kluwer Academic Publishers: Dordrecht, 1999; Vol. C; pp 522–523.

(6) Farrow, C. L.; Juhás, P.; Yang, A.; Billinge, S. J. L.; Yang, X.; Knox, K.; Calamari, J.; Frandsen, B.; Yang, L.; Lee, S.; Chen, Y.; Myers, C.; Zhang, T. Diffpy.Utils. https://github.com/diffpy/diffpy.utils, 2024.

(7) Newville, M.; easyXAFS; Levantino, M.; Schlepuetz, C.; Guenzing, D.; Rakitin, M.; Kim, S.-W.; kalvdans X-rayDB: X-ray Reference Data in SQLite. https://github.com/xraypy/XrayDB, 2024.

(8) Lipp, J.; Banerjee, R.; Patwary, M. F.; Patra, N.; Dong, A.; Girgsdies, F.; Bare, S. R.; Regalbuto, J. R. Extension of Rietveld Refinement for Benchtop Powder XRD Analysis of Ultrasmall Supported Nanoparticles. *Chemistry of Materials* **2022**, *34*, 8091–8111.
